# A fresh look at spinal alignment and deformities: Automated analysis of a large database of 9832 biplanar radiographs

**DOI:** 10.3389/fbioe.2022.863054

**Published:** 2022-07-15

**Authors:** Fabio Galbusera, Tito Bassani, Matteo Panico, Luca Maria Sconfienza, Andrea Cina

**Affiliations:** ^1^ Spine Center, Schulthess Clinic, Zurich, Switzerland; ^2^ IRCCS Istituto Ortopedico Galeazzi, Milan, Italy; ^3^ Department of Chemistry, Materials and Chemical Engineering “Giulio Natta”, Politecnico di Milano, Milan, Italy; ^4^ Department of Biomedical Sciences for Health, Università Degli Studi di Milano, Milan, Italy

**Keywords:** deep learning, sagittal alignment, vertebral landmarks, pelvic incidence, big data, deformity

## Abstract

We developed and used a deep learning tool to process biplanar radiographs of 9,832 non-surgical patients suffering from spinal deformities, with the aim of reporting the statistical distribution of radiological parameters describing the spinal shape and the correlations and interdependencies between them. An existing tool able to automatically perform a three-dimensional reconstruction of the thoracolumbar spine has been improved and used to analyze a large set of biplanar radiographs of the trunk. For all patients, the following parameters were calculated: spinopelvic parameters; lumbar lordosis; mismatch between pelvic incidence and lumbar lordosis; thoracic kyphosis; maximal coronal Cobb angle; sagittal vertical axis; T1-pelvic angle; maximal vertebral rotation in the transverse plane. The radiological parameters describing the sagittal alignment were found to be highly interrelated with each other, as well as dependent on age, while sex had relatively minor but statistically significant importance. Lumbar lordosis was associated with thoracic kyphosis, pelvic incidence and sagittal vertical axis. The pelvic incidence-lumbar lordosis mismatch was found to be dependent on the pelvic incidence and on age. Scoliosis had a distinct association with the sagittal alignment in adolescent and adult subjects. The deep learning-based tool allowed for the analysis of a large imaging database which would not be reasonably feasible if performed by human operators. The large set of results will be valuable to trigger new research questions in the field of spinal deformities, as well as to challenge the current knowledge.

## Introduction

Spinal deformities are common diseases that may have a major impact on the quality of life of affected patients. Prevalences of spinal deformities are relatively high (0.5–5.2% for adolescent idiopathic scoliosis (Kamtsiuris et al., 2007; Konieczny et al., 2013), up to 32% for adult deformities (Schwab et al., 2005)); symptoms depend on the type and severity of the deformity and may include pain, weakness, numbness, dysfunction, breathing disorders, and delayed development. Cosmetic impairments and related psychosocial distress are also common, especially in younger patients (Reichel and Schanz, 2003).

In the last decade, the management of spinal deformities has been largely impacted by the commercial availability of radiographic systems able to capture calibrated, simultaneous biplanar images of the trunk or even of the whole body, such as for example the EOS Imaging System (EOS Imaging, Paris, France). Biplanar radiography allows for an accurate three-dimensional (3D) measurement of quantities that are most commonly assessed in two-dimensional (2D) images (Pasha et al., 2016) such as the spinopelvic parameters (Duval-Beaupere et al., 1992), the sagittal alignment (Barrey et al., 2011; Le Huec et al., 2011) as well as the Cobb angle in the coronal plane (Cobb, 1948), as well as purely 3D parameters not accessible by means of 2D imaging such as vertebral rotations and vectors (Illés et al., 2011; Illés et al., 2019). Such added knowledge of the 3D aspect of the spine shape and curvature is nowadays deemed as crucial for correct pre-operative planning the surgical treatment of the deformity (Illés et al., 2017; Illés et al., 2019). While approximate methods to determine vertebral rotations from simple planar radiographs have been available for decades (Nash and Moe, 1969; Stokes et al., 1986; Perdriolle and Vidal, 1987) and refined similar methods have been recently introduced (Ebrahimi et al., 2019), biplanar imaging offers significantly higher accuracy and reproducibility, even in complex cases (Ilharreborde et al., 2011).

In recent years, the publication of several papers which used machine learning methods to automatically or semi-automatically extract parameters from biplanar radiographs of the spine (Galbusera et al., 2019; Gajny et al., 2019; Vergari et al., 2019; Aubert et al., 2019; Zhang and Li, 2019; Yang et al., 2019) demonstrated the rising interest in the topic and these novel techniques, as well as the need for automatizing a manual process which is time-consuming and relatively user-dependent (Somoskeöy et al., 2012; Bagheri et al., 2018). Our own deep learning tool (Galbusera et al., 2019) proved to be able to perform a fully automated 3D reconstruction of the spine shape as well as to estimate quantities such as spinopelvic parameters, kyphosis and lordosis angles, and coronal Cobb angle with perceptually convincing outcomes for a wide range of clinical scenarios including mild and severe deformities.

In this work, we present an improved version of our deep learning tool, and we used it to process a large dataset of biplanar radiographs of 9,832 non-surgical patients with the aim of describing in detail the statistical distribution of radiological parameters describing the spinal shape and deformities, as well as the correlations and interdependencies between them.

## Materials and methods

### Deep learning model

**FIGURE 1:**
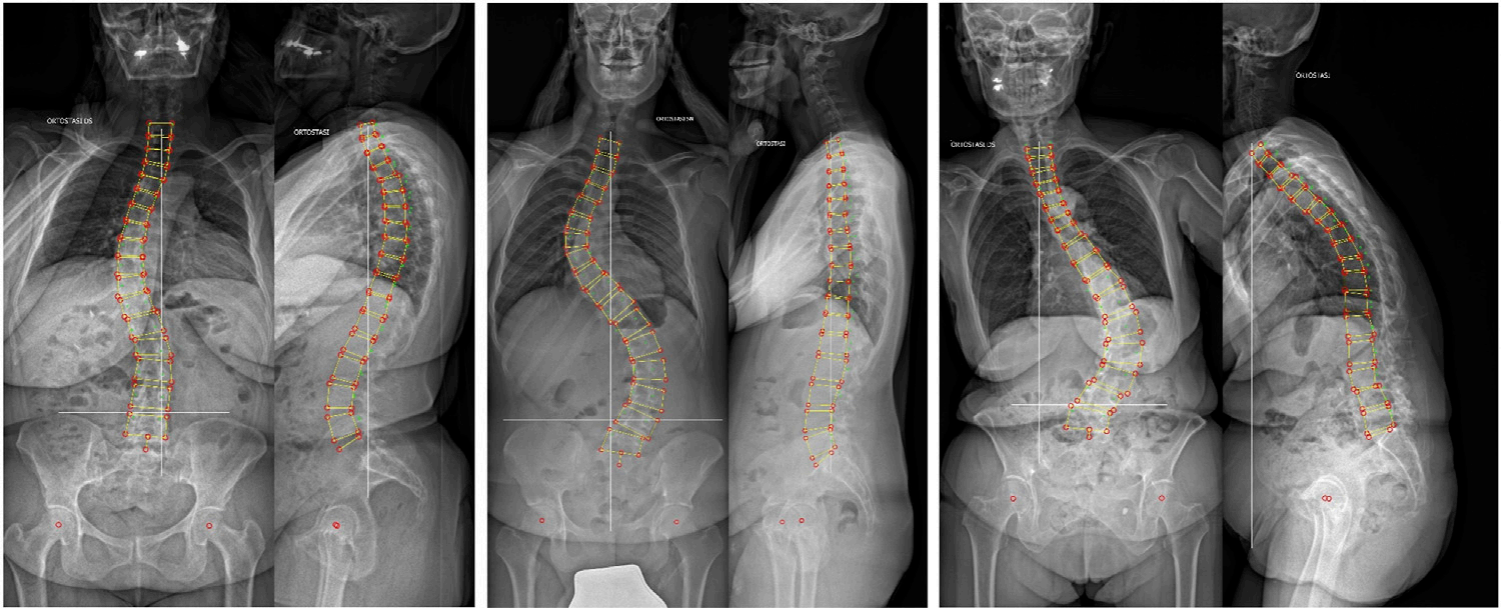
Three randomly selected representative cases of 3D reconstruction of the anatomy of the thoracolumbar spine based on biplanar radiographs of the trunk.

An existing tool able to automatically perform a 3D reconstruction of the anatomy and shape of the thoracolumbar spine based on biplanar radiographs acquired with the EOS Imaging System has been used as the basis for the current study (Galbusera et al., 2019). The deep learning model has been extended by increasing the number of recognized vertebral landmarks from 2 to 10 (Figure 1) and implementing the self-supervision paradigm to improve its performance. In particular, using 10 landmarks allowed for a greater precision in the calculation of the endplate orientation with respect to the previous version, in which we simply considered the line orthogonal to the spline interpolating the two endplate centers. The self-supervised approach allowed exploiting also a set of unlabelled images in the training process in order to increase the model understanding on images. In detail, following the approach presented in Honari et al. (2018) the model produced landmarks localizations equivariant with respect to a set of transformations (rotations and translations) applied to the image. So, for the unlabelled images, if a transformation is applied to an image, the transformed landmarks should be very close to the points obtained by applying the same transformation to landmark coordinates in the original image. In brief, *g(T(I)) ≈ T(g(I))* where *g* is the network, *T* the transformation and *I* the image. As regards the model architecture, while the Differentiable Spatial to Numerical Transform (DSNT) top layer for the regression of the landmark coordinates was kept unaltered from the previous version, the backbone was changed from a simple 7-layer convolutional neural network to a ResNet-34. The size of the training set was extended from 443 to 810.

### Validation

The accuracy of the tool was quantitatively assessed by comparing the automated prediction of various relevant radiological angles with measurements performed by four human observers with an in-house computer-aided tool. The comparison was conducted on three sets of biplanar images of 30 patients which were not included in the training data. The first set referred to patients with no spinal deformity (age 25–70), the second set to patients with adolescent idiopathic scoliosis (age 11–18, coronal Cobb angle 20–65°), while the third set included patients suffering from adult spinal deformities (age 66–85, coronal Cobb angle 3–80°, SVA 1–12 cm). To validate the tool, we counted the number of cases for which the parameter values estimated by the tool fell within the range of the measurements of the four observers, as well as those with an error greater than 5°.

### Patients and radiological parameters

A large set of biplanar radiographs of the full trunk or whole body of 16,228 patients consecutively acquired between 2015 and 2019 at IRCCS Istituto Ortopedico Galeazzi was retrospectively analyzed (Figure 1). All images were acquired in standing in a raised-arm posture to allow for an optimal visualization of the spine in both planes. Non-relevant images were excluded based on the following criteria: 1) age of the subject below 10 years; 2) presence of spinal implants in the images or evidence of non-instrumented spine surgery; 3) neuromuscular and congenital spinal deformities. Patients were stratified based on sex, age group (10–18 years old; 19–44; 45–64; 65–79; 80 or more) as well as on the presence of a spinal deformity, which was defined as a maximal Cobb angle in the coronal plane in the thoracolumbar spine greater than 10° (Lonstein, 1994) and/or a sagittal vertical axis (SVA) greater than 5 cm (Glassman et al., 2005).

For all patients, after using the deep learning model for the prediction of the three-dimensional position of the vertebral landmarks, the values of the following radiological parameters were automatically calculated: spinopelvic parameters (pelvic incidence (PI), pelvic tilt (PT), sacral slope (SS)); lumbar lordosis between L1 and L5 (LL) as well as based on Roussouly’s definition (Roussouly et al., 2003); mismatch between pelvic incidence and lumbar lordosis; thoracic kyphosis between T1 and T12 and between T4 and T12 (TK); maximal Cobb angle in the coronal plane between T1 and L5; SVA; T1-pelvic angle (TPA); in case of scoliosis, maximal vertebral rotation in the transverse plane.

### Data analysis

Scatter plots representing potential correlations of clinical interest between the radiographic parameters were built; linear regression analysis was performed for the same correlations. Analysis of covariance (ANCOVA) was employed to test differences between sexes and age groups in terms of LL, TK, PI, SVA and pelvic incidence-lumbar lordosis mismatch (PI-LL), considering various covariates (age, maximal coronal Cobb angle, PI, LL). Besides, the matrix describing the pairwise Spearman correlation coefficients between various relevant parameters (age, maximal coronal Cobb angle, PI, SS, LL, TK, SVA, TPA, maximal axial rotation) was computed. Finally, the importance of some relevant demographic and radiological parameters (age, sex, PI, SS, LL, TK, SVA, maximal coronal Cobb angle, maximal axial rotation) in determining parameters describing the spinal alignment and possible compensatory mechanisms (LL, TK, SVA, PI-LL, TPA, maximal coronal Cobb angle, maximal axial rotation) were determined by means of a gradient boosted decision tree implemented with the XGBoost *Python* library (https://xgboost.readthedocs.io/).

## Results

### Validation

**FIGURE 2:**
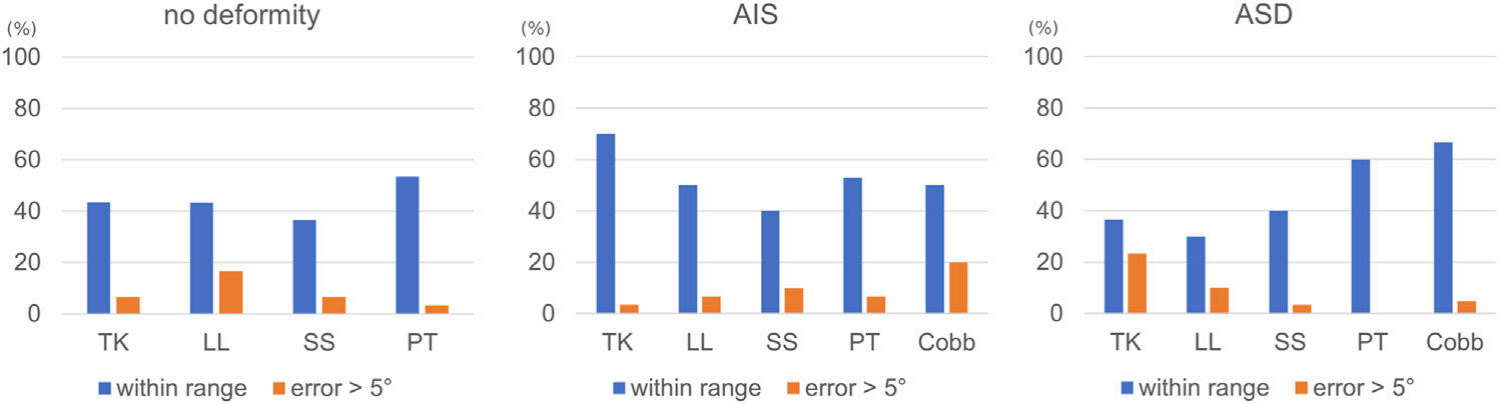
Percentage of cases within the three validation sets (no deformity, adolescent idiopathic scoliosis (AIS), adult spine deformity (ASD)) which were within the range of the manual measurements of four human observers, as well as those with an error greater than 5°.

On average, in 43% of the cases (range 31–72%) the radiological parameters predicted by the automated tool were within the range of the measurements by the four human observers (Figure 2). The cases with error larger than 5° ranged between 0% (pelvic tilt in patients with adult spine deformity) and 20% (maximal coronal Cobb angle in patients with adolescent idiopathic scoliosis).

### Patient population

After the application of the exclusion criteria, biplanar images of 9,832 patients were included in the study. Among these, 5,122 patients had an age between 10 and 18 years, 1,246 between 19 and 44 years, 1,645 between 45 and 64, 1,579 between 65 and 79, and 240 higher than 80 years 5,006 patients were found to have scoliosis, i.e., a maximal coronal Cobb angle higher than 10°, 2,710 of them belonging to the 10–18 years age group. Among the 9,832 patients, 4,428 were found to have no clinically relevant spine deformities, i.e., Cobb angle lower than 10° and SVA lower than 5 cm. Table 1 shows range, mean and median values of several radiological parameters within the population.

**TABLE 1:** Range, mean and median values of radiological parameters of the population.

parameter	unit	range	mean	median
PI	degrees	3–89	49	49
SS	degrees	2–75	37	37
L1-L5 lordosis	degrees	-15–74	40	42
T4-T12 kyphosis	degrees	-17–79	39	38
SVA	cm	-9.7–27.6	0.3	-0.6
maximal coronal Cobb angle	degrees	1–106	15	10
maximal axial rotation	degrees	0–47	7	6

### Sagittal alignment

**FIGURE 3:**
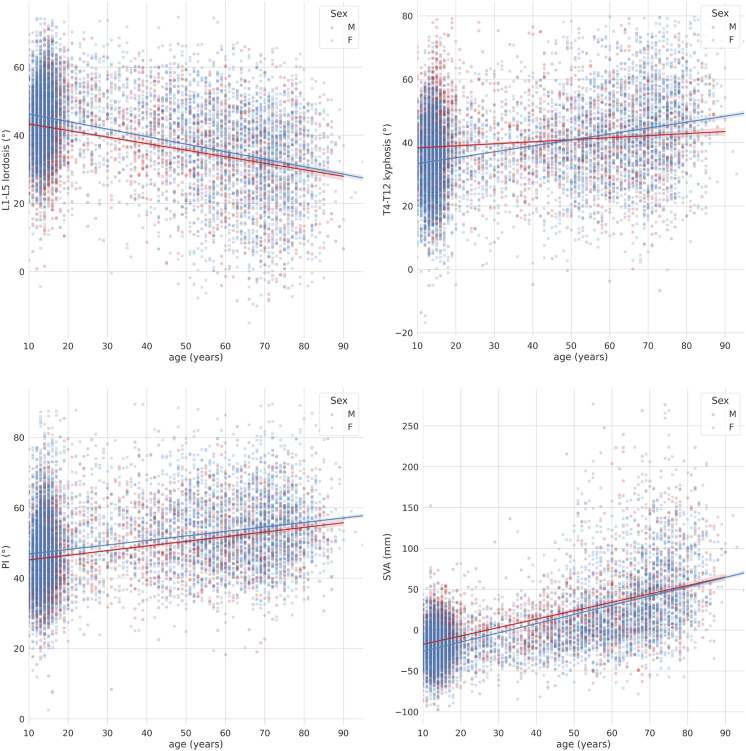
Scatter-regression plots describing the association between age and sagittal parameters, with patients stratified based on sex (“M”: males; “F”: females). First row: L1-L5 lumbar lordosis (left) and T4-T12 thoracic kyphosis (right); second row: pelvic incidence (PI) (left) and sagittal vertical axis (SVA) (right).

The radiological parameters describing the sagittal alignment of the spine were found to be highly interrelated with each other, as well as dependent on age, while sex had relatively minor importance (Figure 3). Nevertheless, all performed ANCOVA analyses to test differences between age groups as well as sex in terms of sagittal parameters showed statistical significance regardless of the magnitude of such differences, due to high numerosity of the sample. The full set of charts describing the associations between the radiological parameters is reported in the Supplementary Material.

**FIGURE 4:**
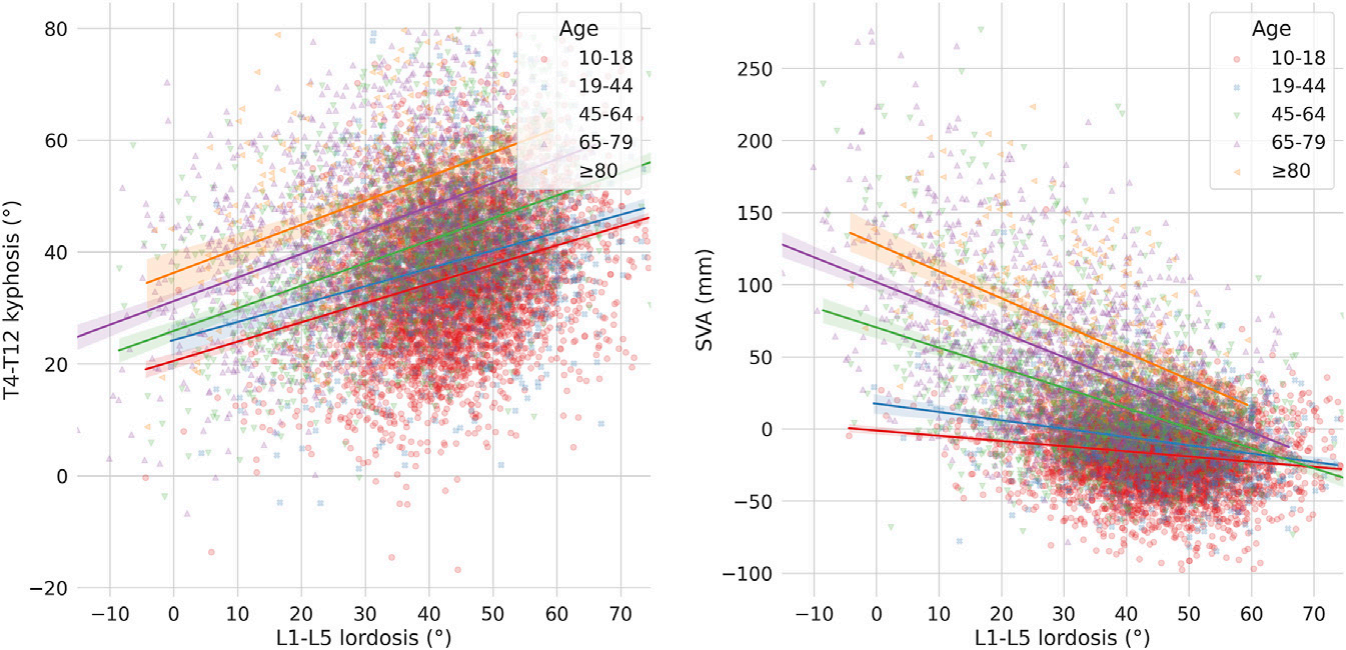
Scatter-regression plots describing the association between L1-L5 lordosis and: T4-T12 kyphosis (left), sagittal vertical axis (SVA) (right), with patients stratified based on age groups.

The L1-L5 lumbar lordosis was associated with both the thoracic kyphosis and SVA (Figure 4). While the increase in thoracic kyphosis with respect to the change in lumbar lordosis, i.e., the slope of the regression curve, was weakly dependent on the age, older subjects had markedly higher kyphosis with respect to younger ones being the lumbar lordosis equal. In contrast, the slope of the regression curve describing SVA with respect to the lumbar lordosis was dependent on the age, i.e., older subjects had higher changes in SVA being the change in lumbar lordosis equal, demonstrating the importance of age in determining how the global sagittal balance responds to a decrease in lumbar lordosis.

**FIGURE 5:**
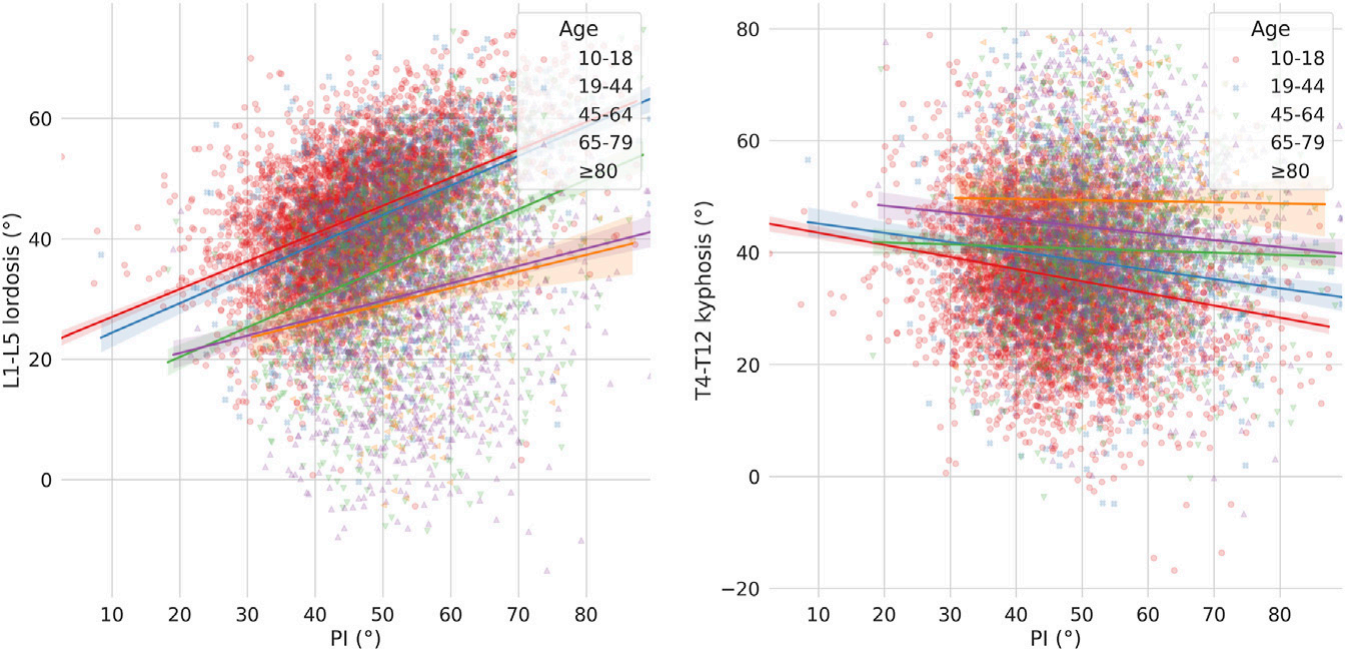
Scatter-regression plots describing the association between pelvic incidence (PI) and: L1-L5 lordosis (left), T4-T12 kyphosis (right), with patients stratified based on age groups.

A strong association between PI and lumbar lordosis was found, while the thoracic kyphosis showed a less clear correlation with PI (Figure 5). Regarding the lumbar lordosis, while all age groups showed a positive correlation, the slope of the regression lines was higher for younger subjects, with minimal differences between the 10–18 and 19–44 age groups as well as for subjects older than 65 years. The thoracic kyphosis tended to decrease with increasing PI, with a greater effect for the younger subjects.

**FIGURE 6:**
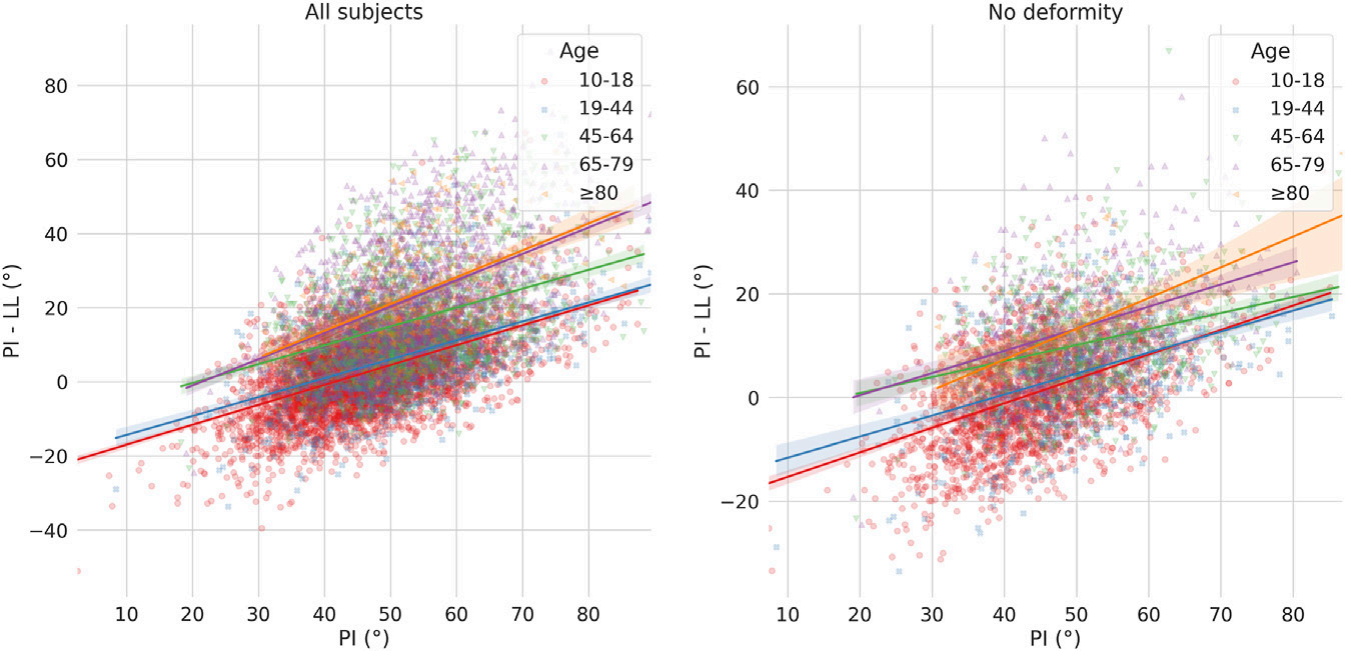
Scatter-regression plots describing the association between pelvic incidence (PI) and pelvic incidence-lumbar lordosis mismatch (PI-LL), considering all patients (left) and only those with no spinal deformities (right). Patients are stratified based on age groups.

The pelvic incidence-lumbar lordosis mismatch was found to be dependent on PI, as well as on age (Figure 6). In general, PI-LL values increased with increasing PI, with the slope of the regression line showing only minor differences among the age groups. The same trend was observed even after excluding subjects with spinal deformities.

### Scoliosis

**FIGURE 7:**
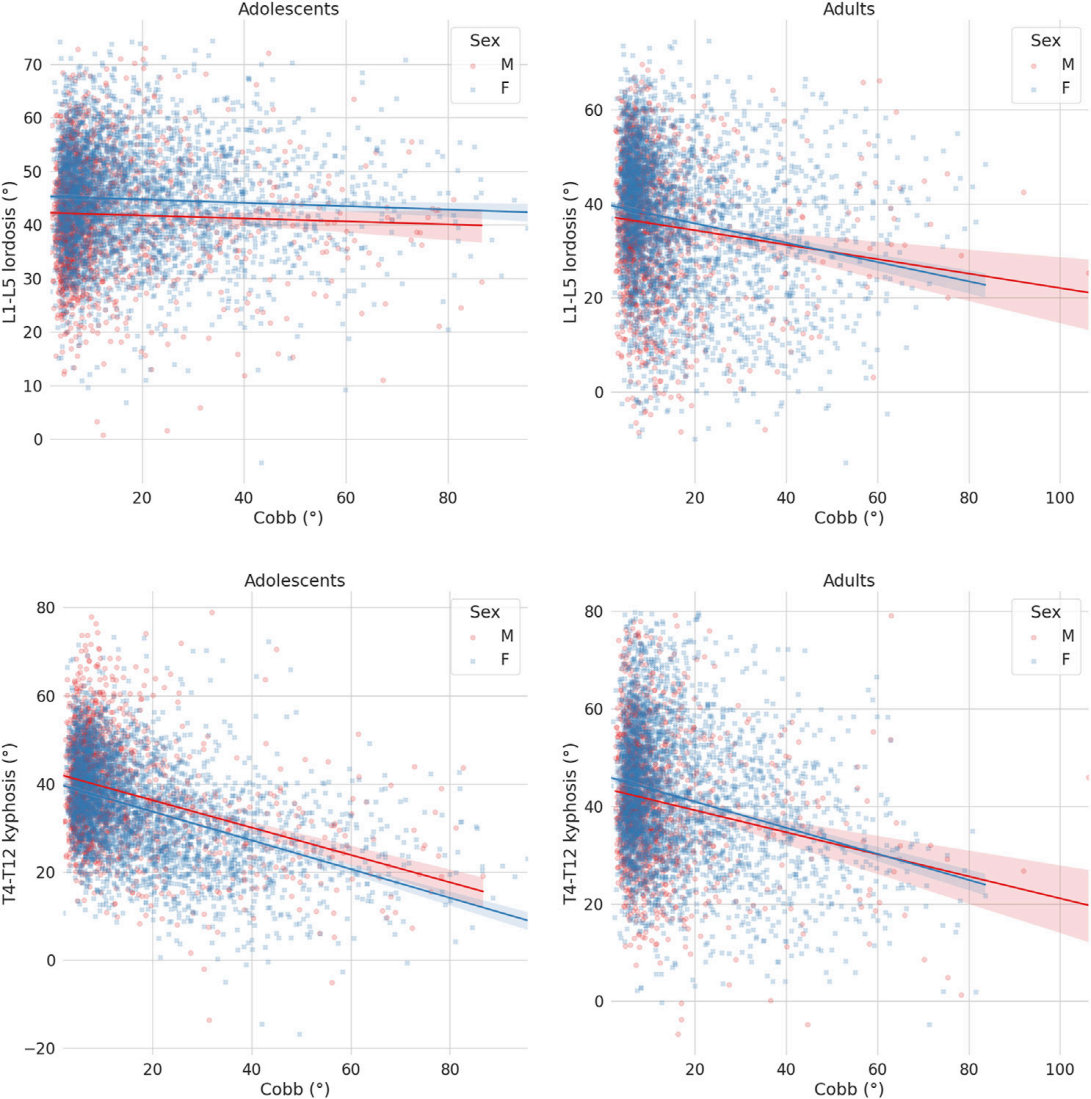
Scatter-regression plots describing the association between the maximal Cobb angle in the coronal plane and sagittal parameters, with patients stratified based on sex (“M”: males; “F”: females). First row: L1-L5 lordosis in adolescents (left) and adults (right); second row: T4-T12 kyphosis in adolescents (left) and adults (right).

The severity of scoliosis, as described by the maximal Cobb angle in the coronal plane, had a distinct association with the sagittal alignment in adolescent subjects with respect to the older patients (Figure 7). Similar to the sagittal parameters, all ANCOVA analyses (i.e. effect of sex and age group considering the coronal Cobb angle as covariate) showed statistical significance, even if the magnitude of such effects was small. While the Cobb angle was weakly associated with a change in lumbar lordosis in the group of young subjects, the lordosis showed a marked decrease for more severe curves in adults. On the contrary, the thoracic kyphosis showed a clear tendency toward a decrease with the severity of the scoliotic curve in both adolescents and adults, with approximately similar behaviours.

### Correlation between parameters

**FIGURE 8:**
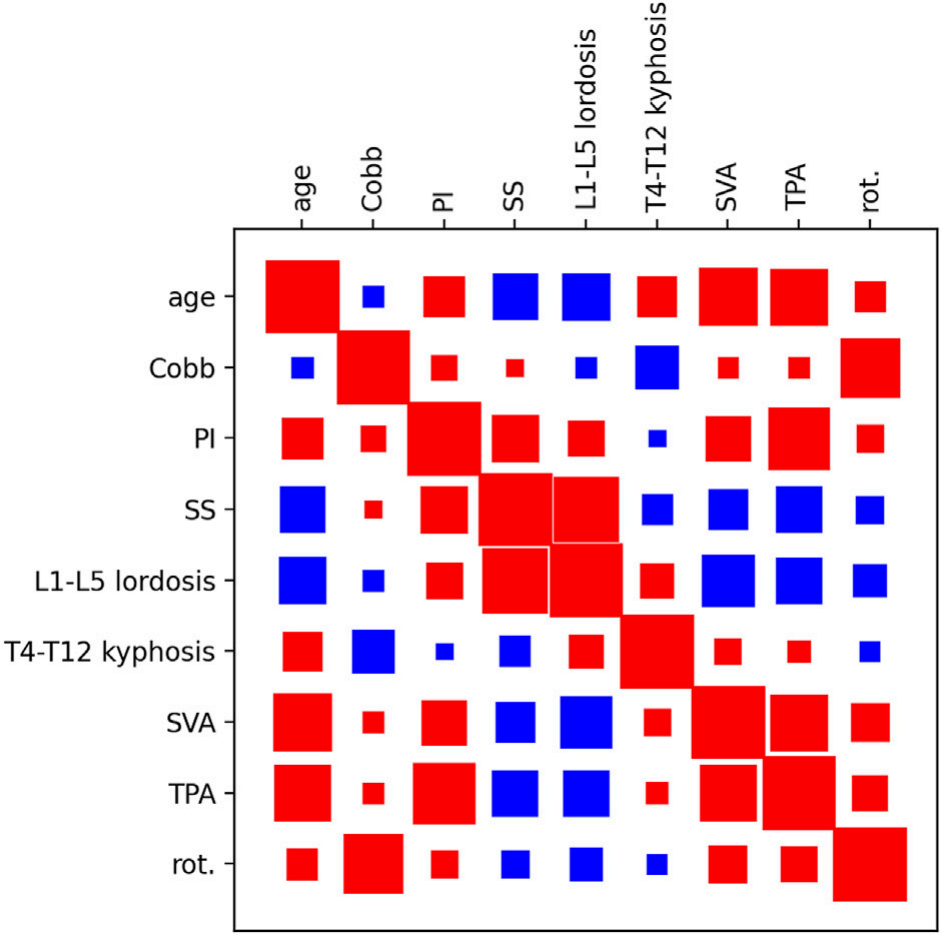
Hinton diagram showing the pairwise correlation between various parameters (age, maximal coronal Cobb angle (Cobb), PI, SS, L1-L5 lordosis, T4-T12 kyphosis, SVA, T1-pelvic angle (TPA), maximal vertebral rotation in the transverse plane (rot.). Red indicates a positive correlation, blue a negative correlation. The size of the square indicates the strength of the correlation.

The matrix showing the Spearman correlation coefficients between various parameters confirmed the strong interdependence between them, as well as the findings widely documented in the literature and highlighted in the previous paragraphs (Figure 8). Higher age is associated with lower LL, higher TK, SVA and TPA, as well *p*I. Higher PI is correlated with higher SS, LL, SVA and TPA, whereas it had a minor correlation with TK. SS and LL show a high correlation, as well as the maximal Cobb angle and the maximal rotation in the axial plane.

### Importance of the predictors

**FIGURE 9:**
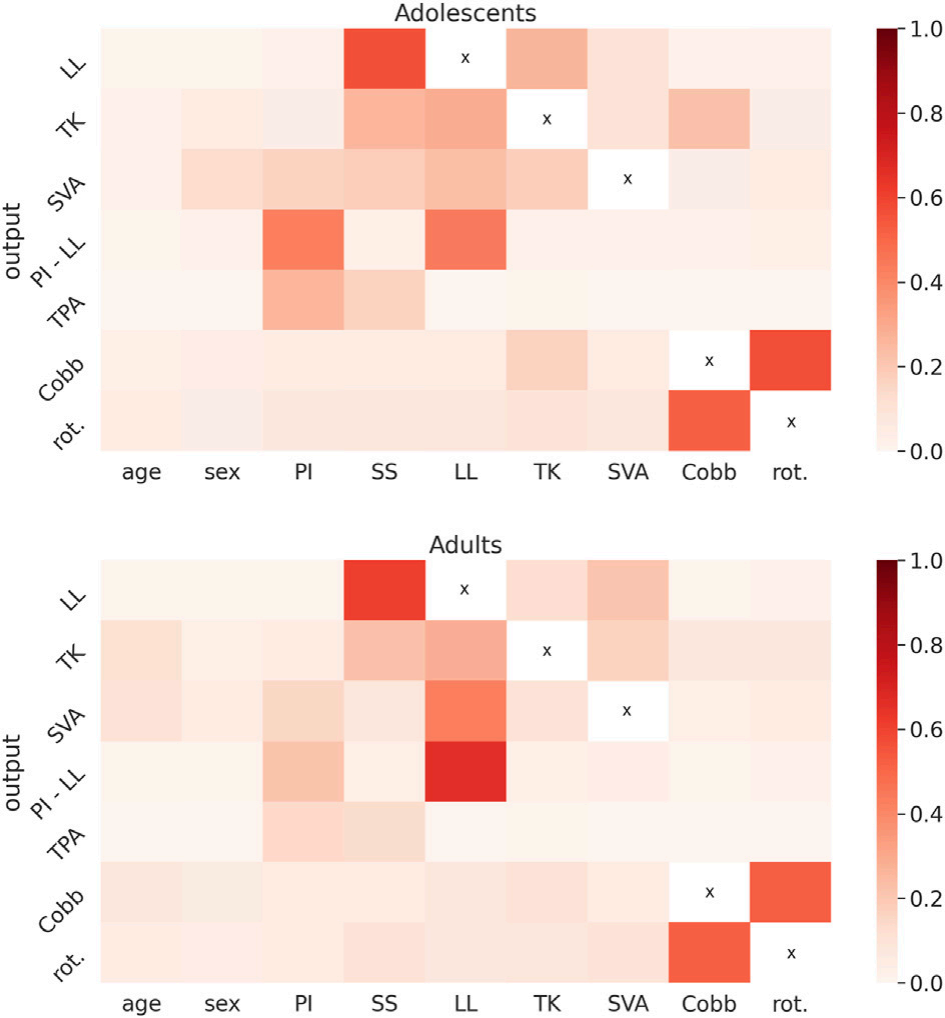
Heatmaps showing the importance of the predictors (age, sex, pelvic incidence (PI), sacral slope (SS), L1-L5 lordosis (LL), T4-T12 kyphosis (TK), sagittal vertical axis (SVA), maximal coronal Cobb angle (Cobb), maximal vertebral rotation in the transverse plane (rot.)) in determining a set of radiological parameters (LL, TK, SVA, pelvic incidence-lumbar lordosis mismatch (PI-LL), T1-pelvic angle (TPA), Cobb, rot.) obtained with the XGBoost model, for the adolescent (top) and for the adult subjects (bottom).

The XGBoost model showed that the importance of the demographic and radiological parameters in determining the spinal alignment did not exhibit major differences between adolescent and adult subjects (Figure 9). The spinopelvic parameters, especially the sacral slope which includes both anatomical and postural information, together with the lumbar lordosis had as expected high importance in determining other sagittal parameters. In adults, SVA (that describes the global sagittal balance) was mostly determined by the lumbar lordosis, while in adolescents all sagittal parameters contributed to it. The strongest predictor for the coronal Cobb angle was the maximal rotation in the transverse plane, while no radiological parameter describing the sagittal alignment (with the partial exception of the thoracic kyphosis for the adolescent subjects) seemed to play a major role in its determination.

## Discussion

This paper presented the largest retrospective analysis to date of a database of biplanar radiographs of the full spine of non-operated patients suffering from spinal deformities covering a wide range of severities, which was conducted by using automated software based on a state-of-the-art deep learning method. The use of a tool not requiring the manual intervention of an operator allowed the analysis of a large number of images with high reproducibility and robustness, and revealed findings that are difficult to observe in smaller clinical studies.

The deep learning tool used in this paper builds on an existing model (Galbusera et al., 2019), which was refined from a methodological point of view and extended by approximately doubling the number of images used to train the neural network. To our knowledge, no other model able to automatically extract vertebral locations and orientations of all thoracolumbar vertebrae from biplanar images of the trunk is currently available, but several research groups are developing similar tools and the outlook is very promising. Among the several papers published recently, Weng et al. presented a tool for the automated calculation of the SVA in lateral images (Weng et al., 2019), Yeh et al. were able to accurately predict the location of vertebral landmarks in lateral images (Yeh et al., 2021), and Korez and others developed a deep learning model for the automatic calculation of the spinopelvic parameters with performances comparable to that of human observers (Korez et al., 2020). As a matter of fact, while it would be premature to conclude that software based on artificial intelligence can replace physicians in the radiographic analysis of spinal deformities, it is nonetheless evident that such a possibility is rapidly becoming more and more realistic.

The amount of information that can be revealed by the automated analysis of a large radiographic dataset of patients in a wide range of age and clinical scenarios is immense and goes beyond the scope of a single research paper. Nevertheless, we decided to release a large set of charts as Supplementary Material, which can serve as a starting point for further studies in the field, by triggering new research questions or challenging existing knowledge. An example of such an issue that deserves further investigation is the pelvic incidence-lumbar lordosis mismatch, which has recently gained a lot of attention as a target for the surgical correction of sagittal imbalance in adult patients (Schwab et al., 2013). High values of PI-LL, i.e. significant losses of lumbar lordosis, were found to be associated with poor health-related quality of life and disability (Glassman et al., 2005; Merrill et al., 2017). A value of 10° is frequently considered as a threshold indicating a high mismatch, and is therefore considered as a target value to be achieved in order to obtain a good surgical correction with a low risk of complications (Schwab et al., 2010; Rothenfluh et al., 2015). Nevertheless, to our knowledge the statistical distribution of PI-LL in a large population of balanced subjects (SVA <5 cm), either in presence of compensatory mechanisms or not, has never been conducted and therefore the selection of the threshold value may appear to be rather arbitrary. In a previous study, Hyun and coworkers already noted, although on a smaller cohort of 150 elderly volunteers, that the ideal values of PI-LL are inconsistent and positively correlated with PI (Hyun et al., 2019), in agreement with the current data and demonstrating that the issue deserves a deeper investigation. It should also be noted that the present large-scale analysis would constitute a valuable basis for such a study, for example by performing a multivariate analysis with is indeed currently being conducted.

This study is not without limitations. First, the data collection was retrospective and detailed information about the indications for imaging were not accessible; the only available data about the patients were age and sex. The exclusion criteria (previous spine surgery, neuromuscular or congenital deformities) were applied exclusively based on a visual inspection of the images, which might not be obvious for example in the case of non-instrumented surgery. While the version of the deep learning model used in this paper includes several improvements with respect to the original version (Galbusera et al., 2019) and was trained on a larger dataset, some degree of error in the localization of the landmarks cannot be excluded especially in the case of major deformities, as documented in the validation against human raters. Such results should be evaluated accounting for the relative lack of reliability of measurements performed by humans; indeed, considering the Cobb angle of scoliosis as a reference, an average error of 3.7–7.2° when using manual tools (Morrissy et al., 1990; Wang et al., 2018) and of 1.7–1.9° with computer-aided systems (Hurtado-Avilés et al., 2022) were reported, demonstrating that further improvements are necessary prior to a generalized clinical use of automated measurement systems. Finally, as mentioned above detailed statistical analyses addressing specific research questions were not conducted, since this paper aimed at presenting the dataset in a descriptive form with a methodological focus on the deep learning technique used for the evaluation of the images.

In conclusion, the present deep learning-based tool allowed for the analysis of a large imaging database which would not be reasonably feasible in the frame of a research project, if performed by human operators. The large set of results here reported will be valuable as a reference for future studies as well as to trigger new research questions in the field of spinal deformities, or to challenge the current knowledge. We believe that algorithms based on artificial intelligence will determine an enormous increase in the availability of data extracted from radiological imaging in the next future, benefiting both spine research and care.

## Data Availability

The raw data supporting the conclusions of this article will be made available by the authors, without undue reservation.
