# Supplementary material for: A fresh look at spinal alignment and deformities: Automated analysis of a large database of 9832 biplanar radiographs
Source: Front Bioeng Biotechnol. 2022 Jul 15;10:863054. doi: 10.3389/fbioe.2022.863054 (PMC9335010; doi:10.3389/fbioe.2022.863054)
Supplement: Supplementary file 1 [file DataSheet1.PDF]

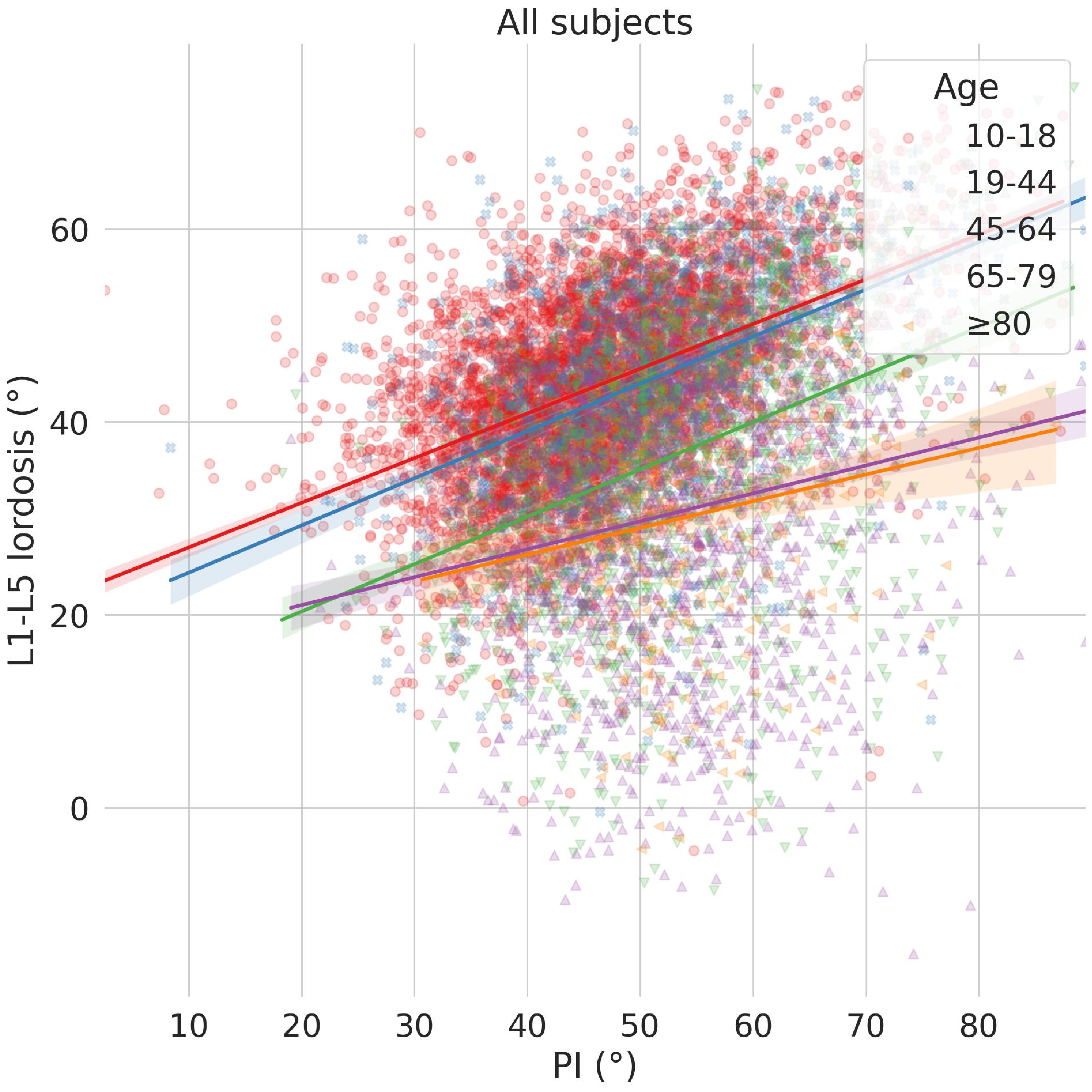
All subjects
Age
10-18
19-44
45-64
65-79
≥80
L1-L5 lordosis (°)
PI (°)
0
20
40
60
10
20
30
40
50
60
70
80

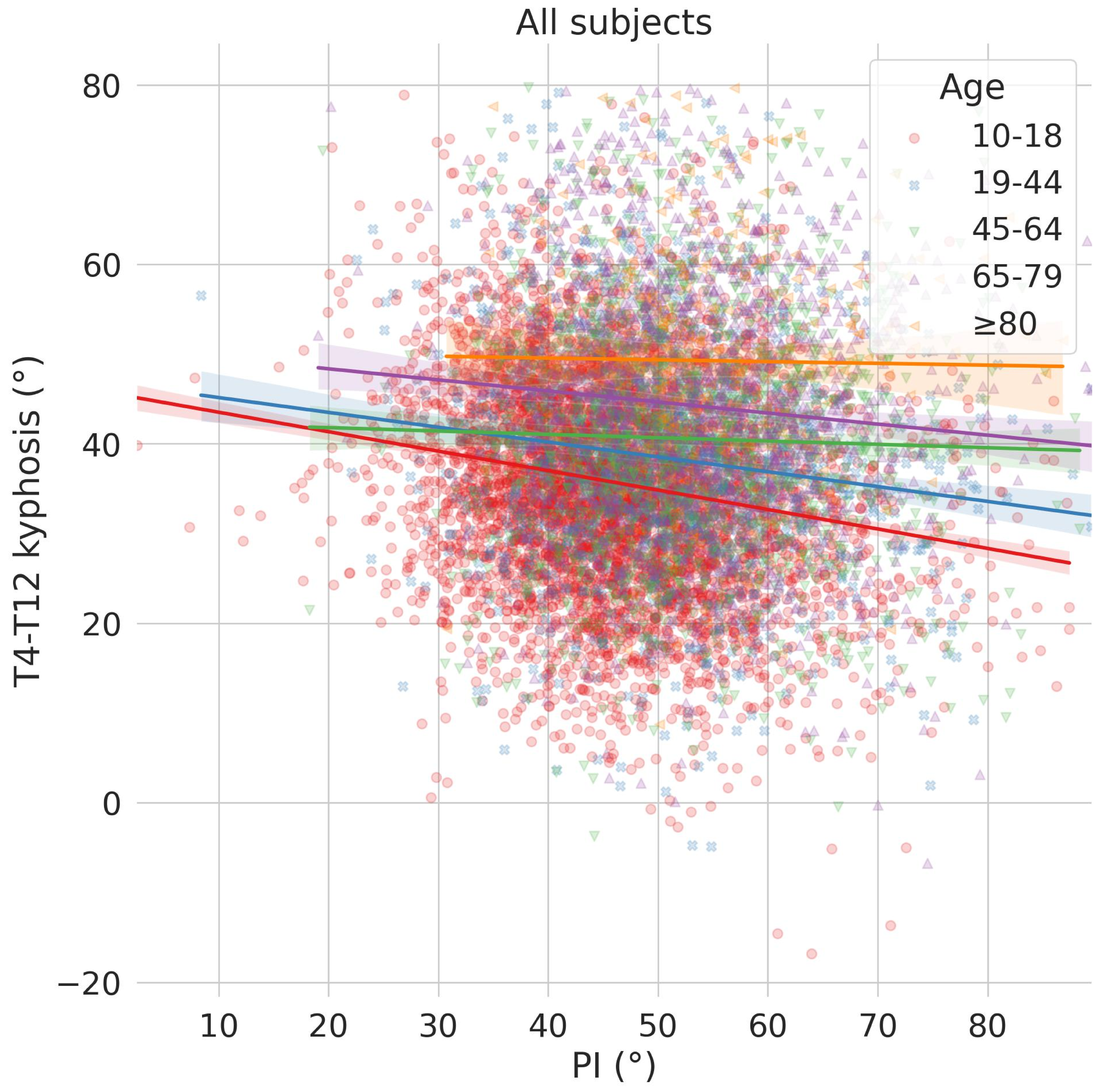
All subjects
Age
10-18
19-44
45-64
65-79
≥80
T4-T12 kyphosis (°)
PI (°)
80
60
40
20
0
−20
10
20
30
40
50
60
70
80

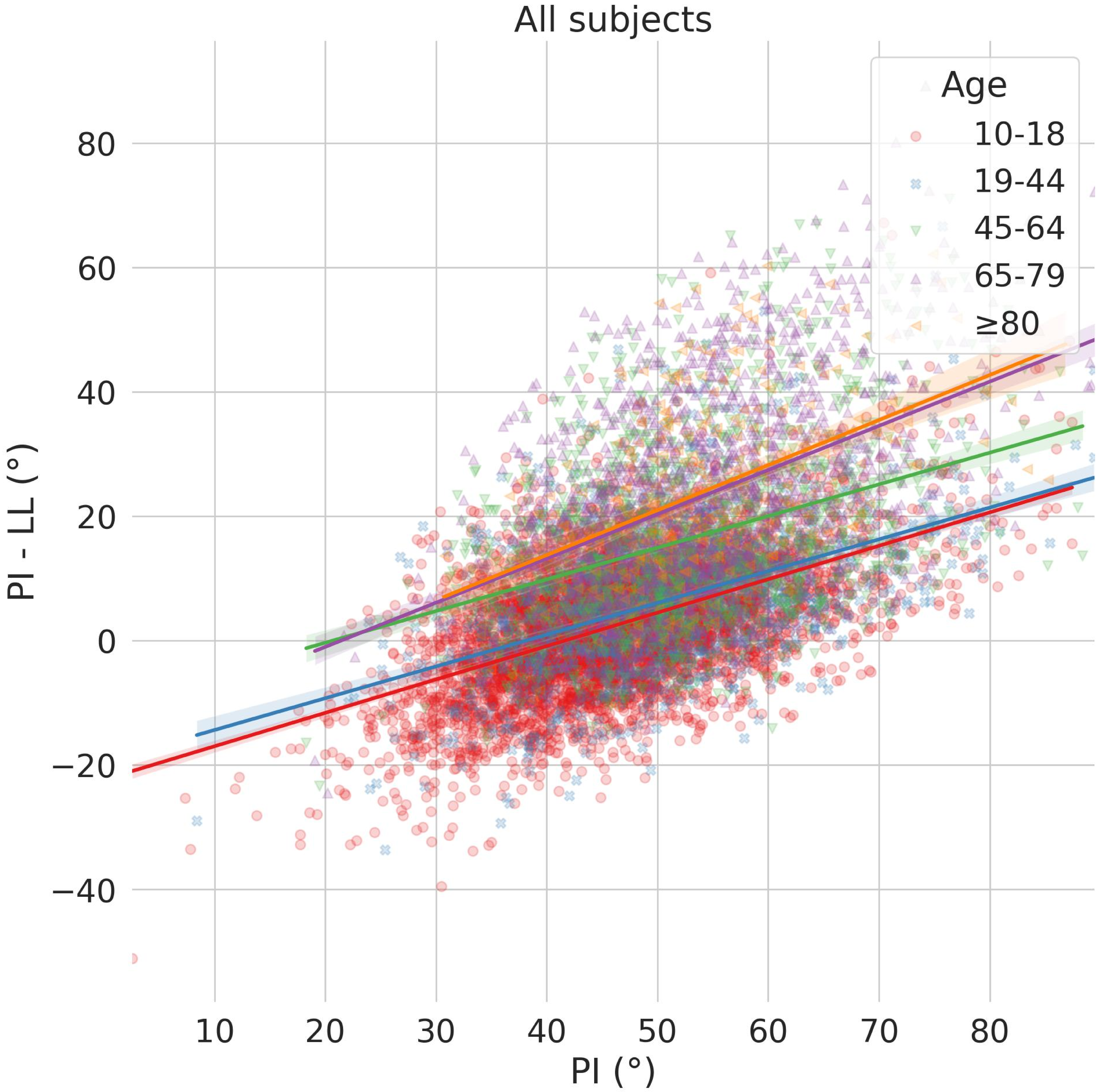

All subjects
Age
10-18
19-44
45-64
65-79
≥80
PI - LL (°)
PI (°)
80
60
40
20
0
−20
−40
10
20
30
40
50
60
70
80

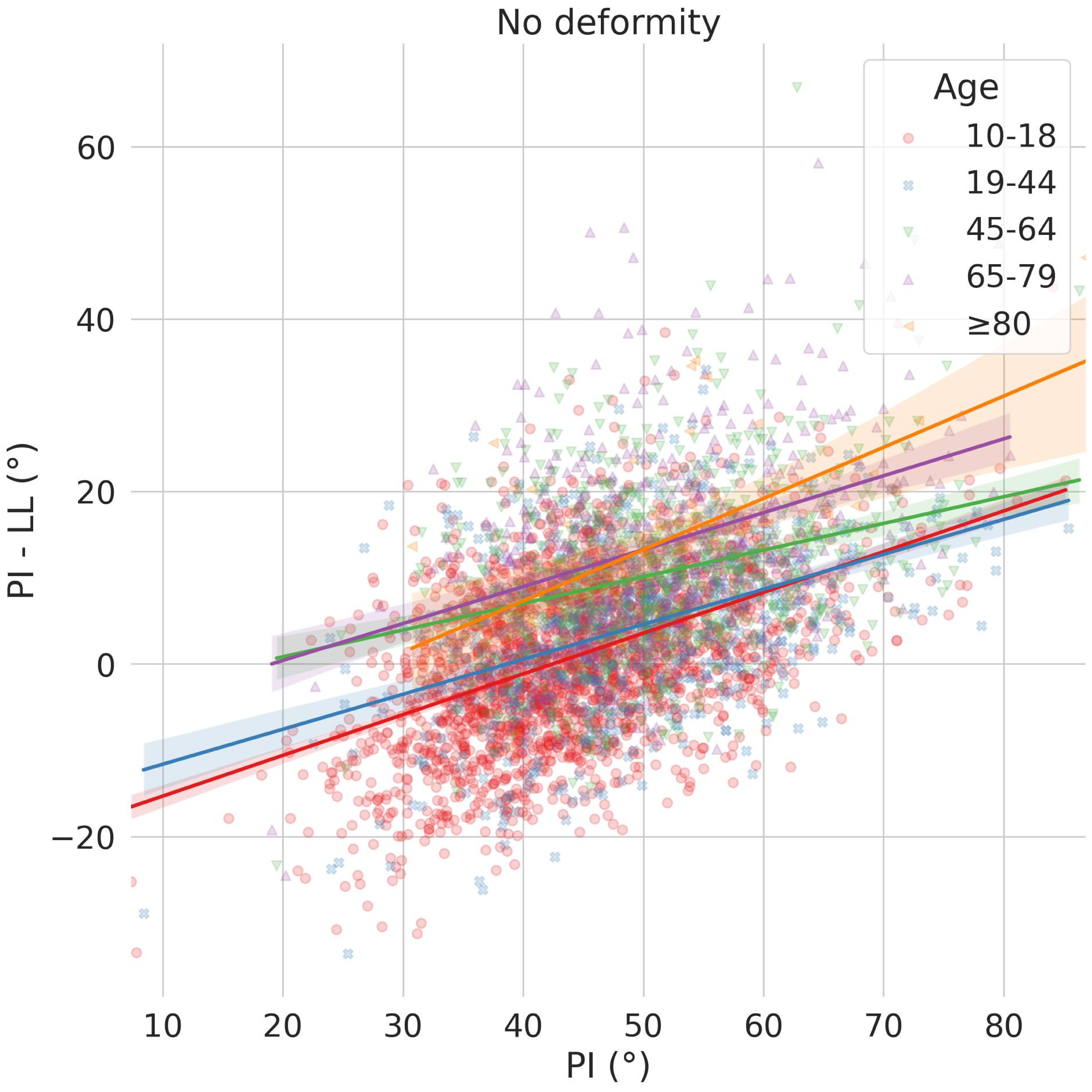

No deformity
Age
10-18
19-44
45-64
65-79
≥80
60
40
20
0
−20
PI - LL (°)
10
20
30
40
50
60
70
80
PI (°)

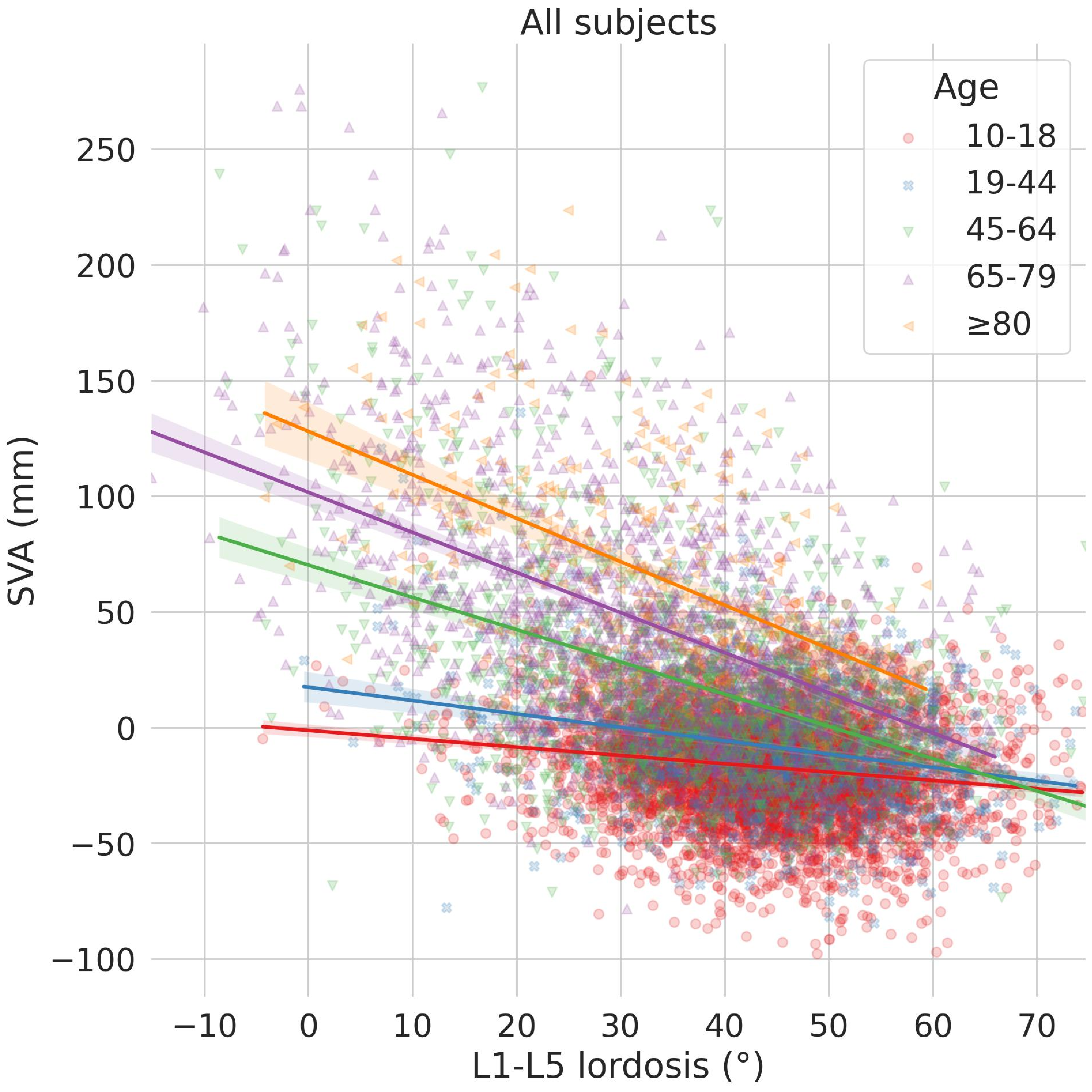
All subjects
Age
10-18
19-44
45-64
65-79
≥80
SVA (mm)
250
200
150
100
50
0
−50
−100
−10
0
10
20
30
40
50
60
70
L1-L5 lordosis (°)

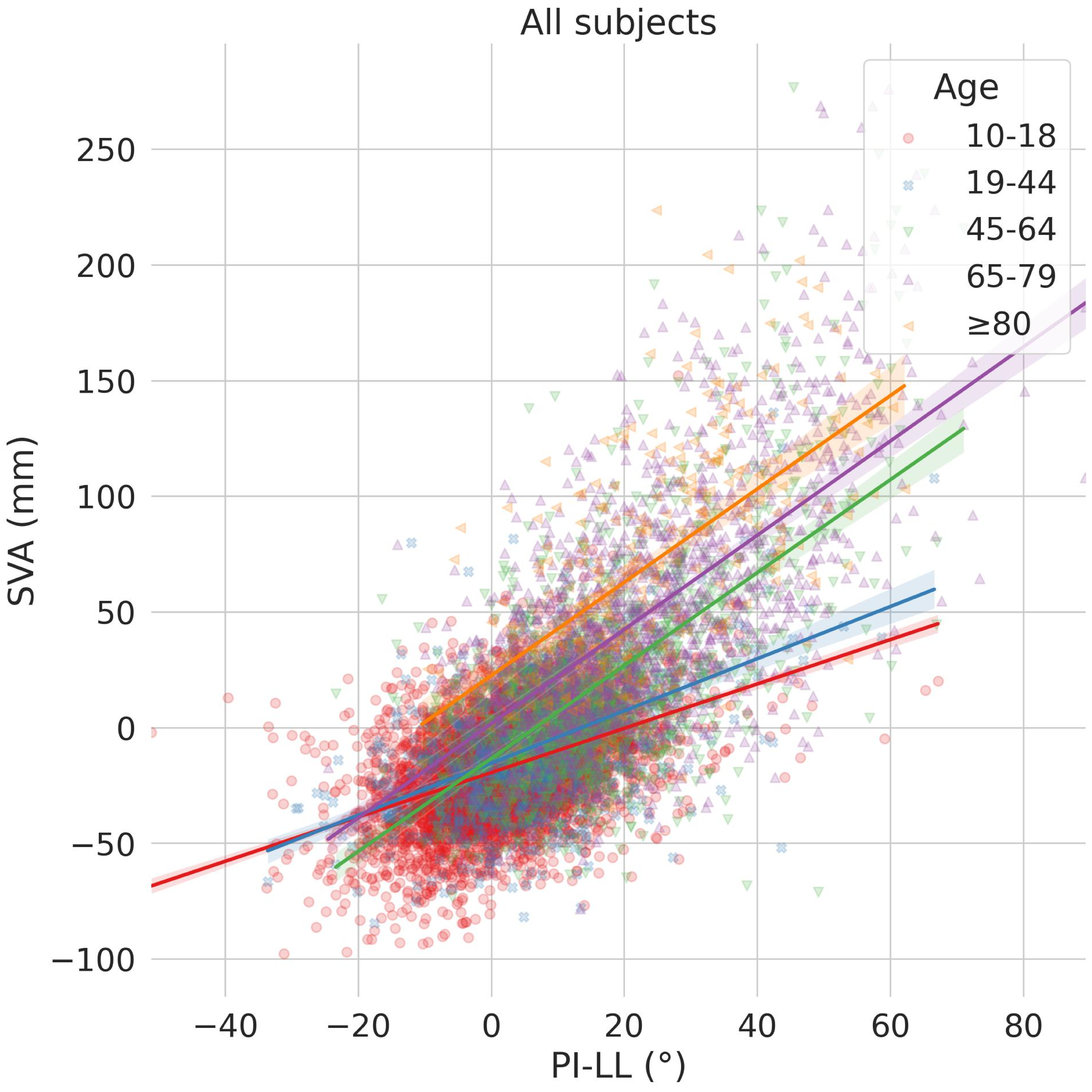
All subjects
Age
10-18
19-44
45-64
65-79
≥80
SVA (mm)
PI-LL (°)
250
200
150
100
50
0
−50
−100
−40
−20
0
20
40
60
80

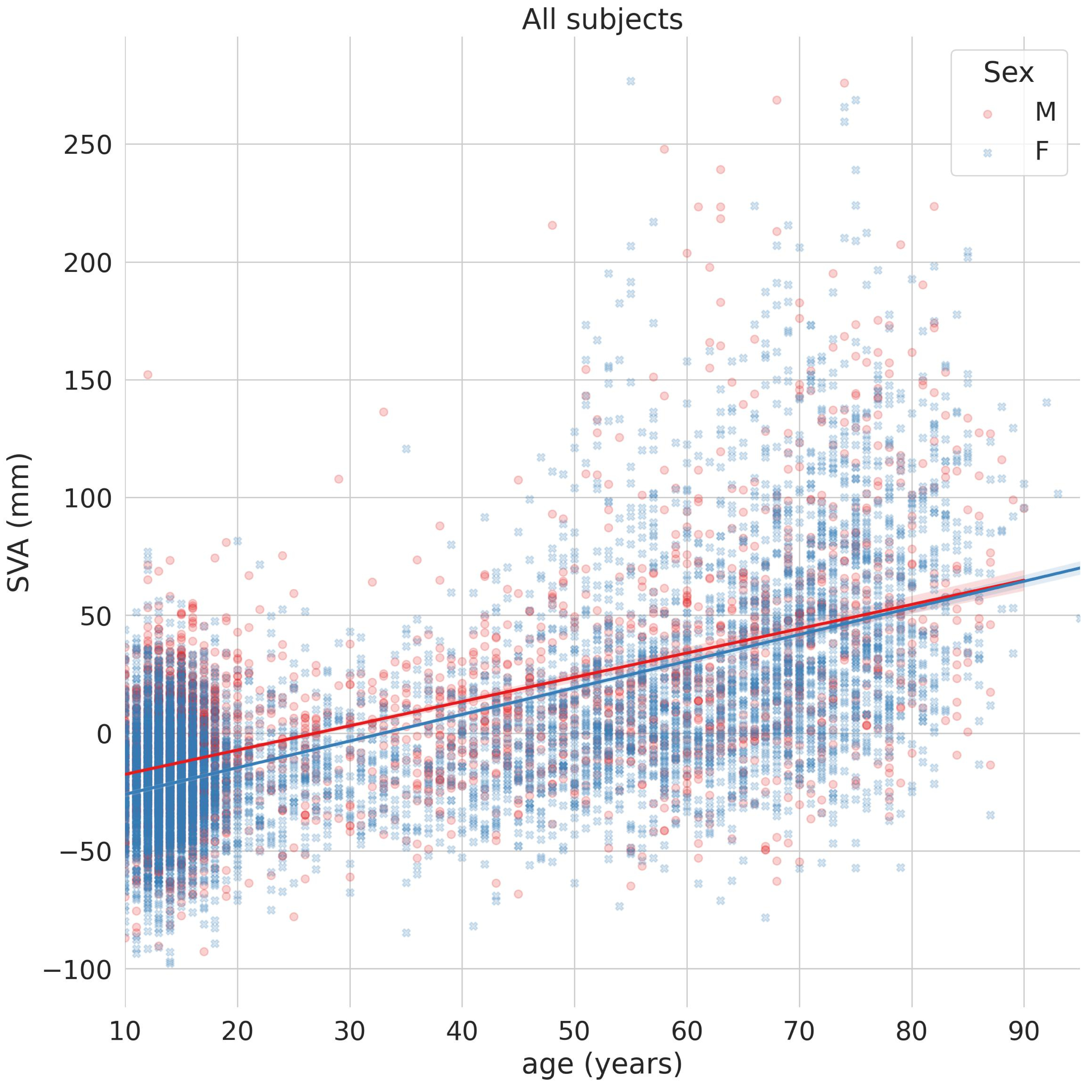
All subjects
Sex
M
F
SVA (mm)
age (years)
250
200
150
100
50
0
−50
−100
10
20
30
40
50
60
70
80
90

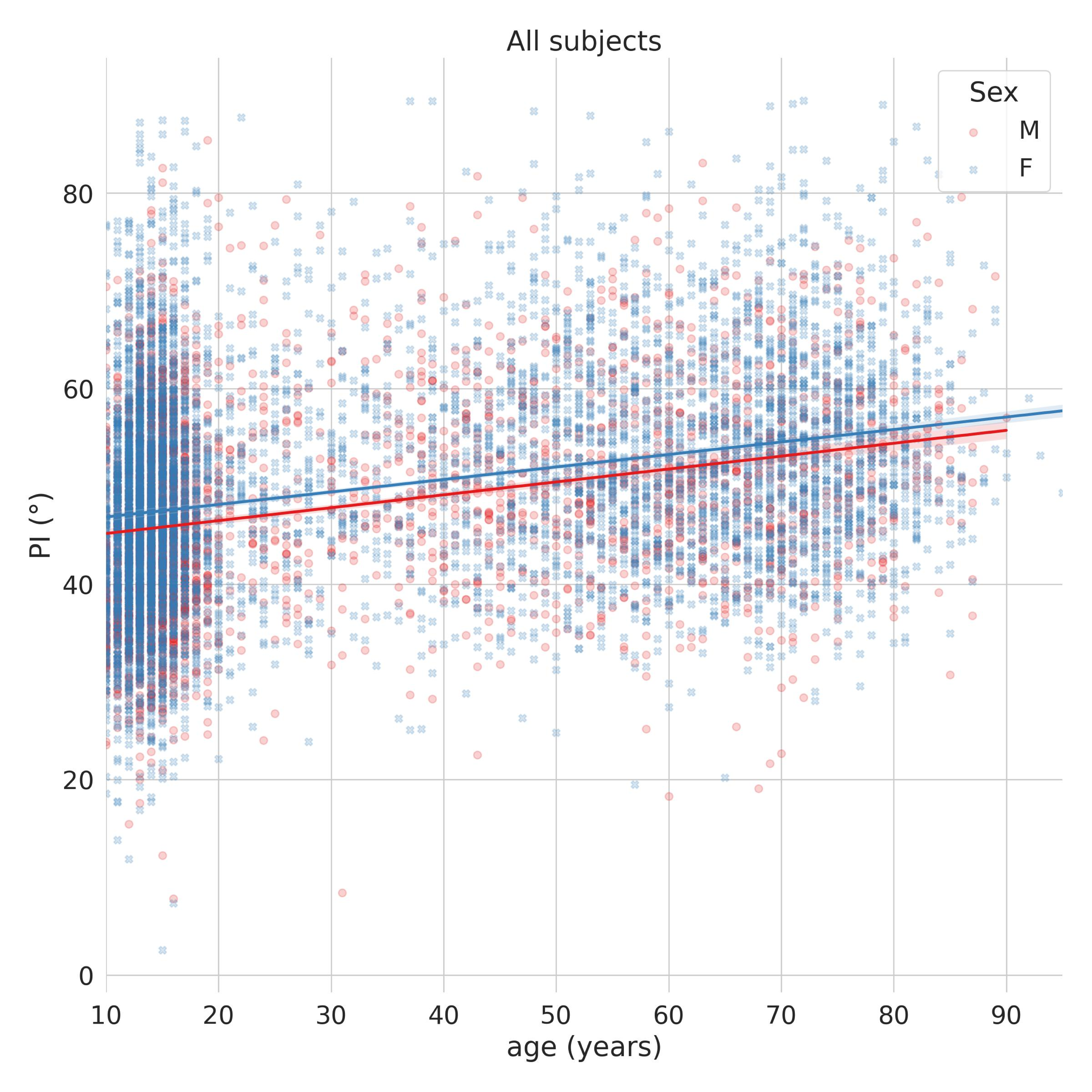

All subjects
Sex
M
F
PI (°)
0
20
40
60
80
10
20
30
40
50
60
70
80
90
age (years)

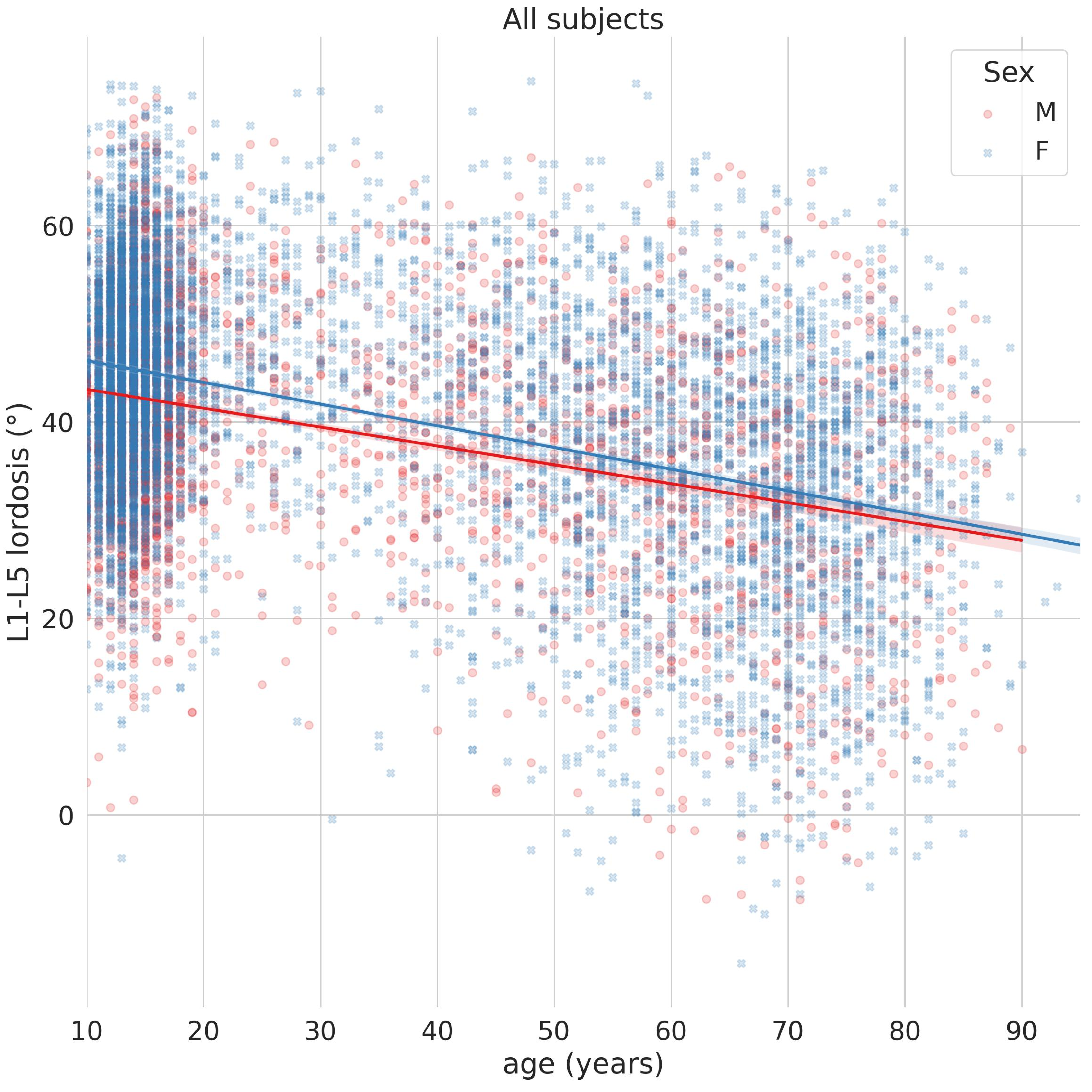

All subjects
Sex
M
F
L1-L5 lordosis (°)
age (years)
60
40
20
0
10
20
30
40
50
60
70
80
90

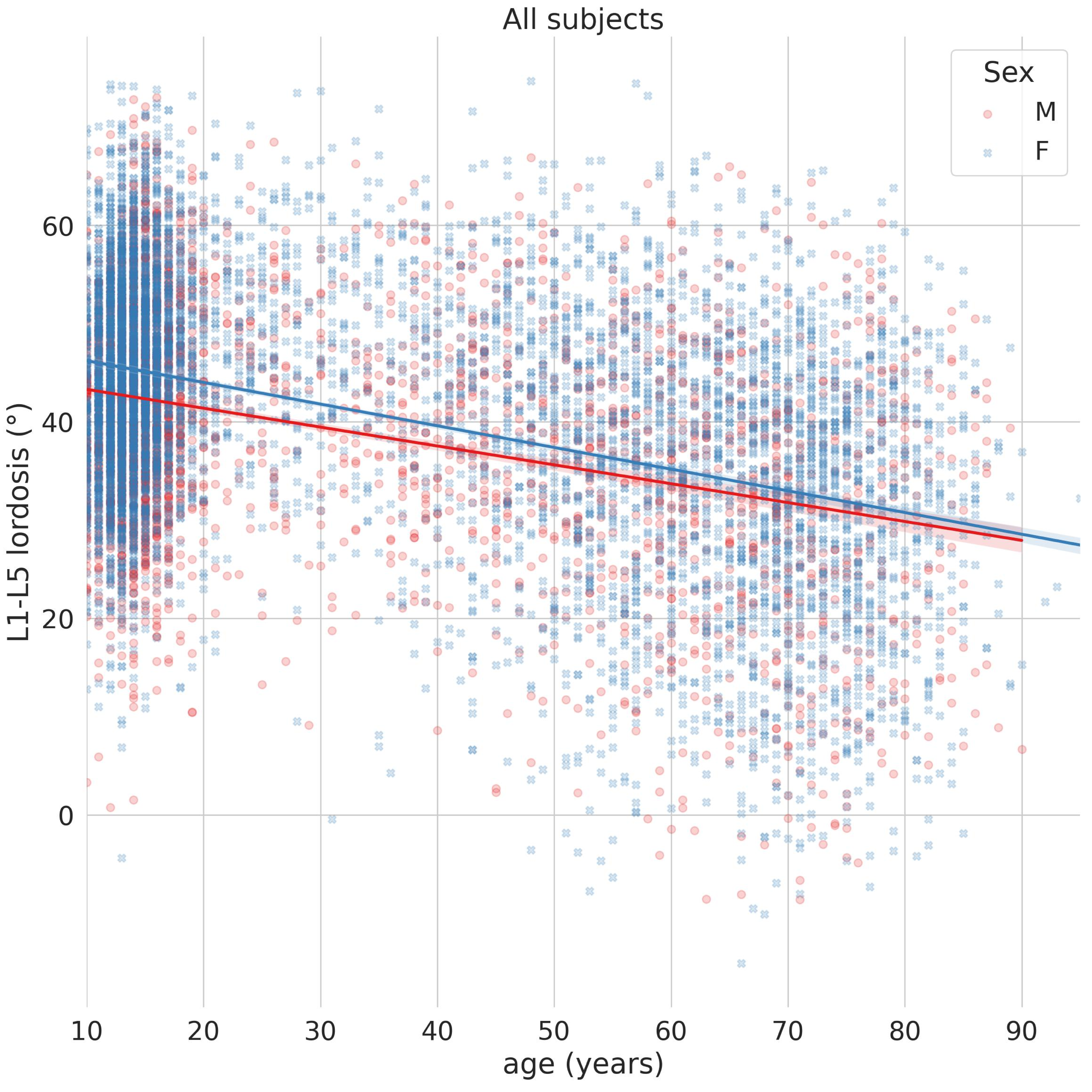
All subjects
Sex
M
F
L1-L5 lordosis (°)
0
20
40
60
10
20
30
40
50
60
70
80
90
age (years)

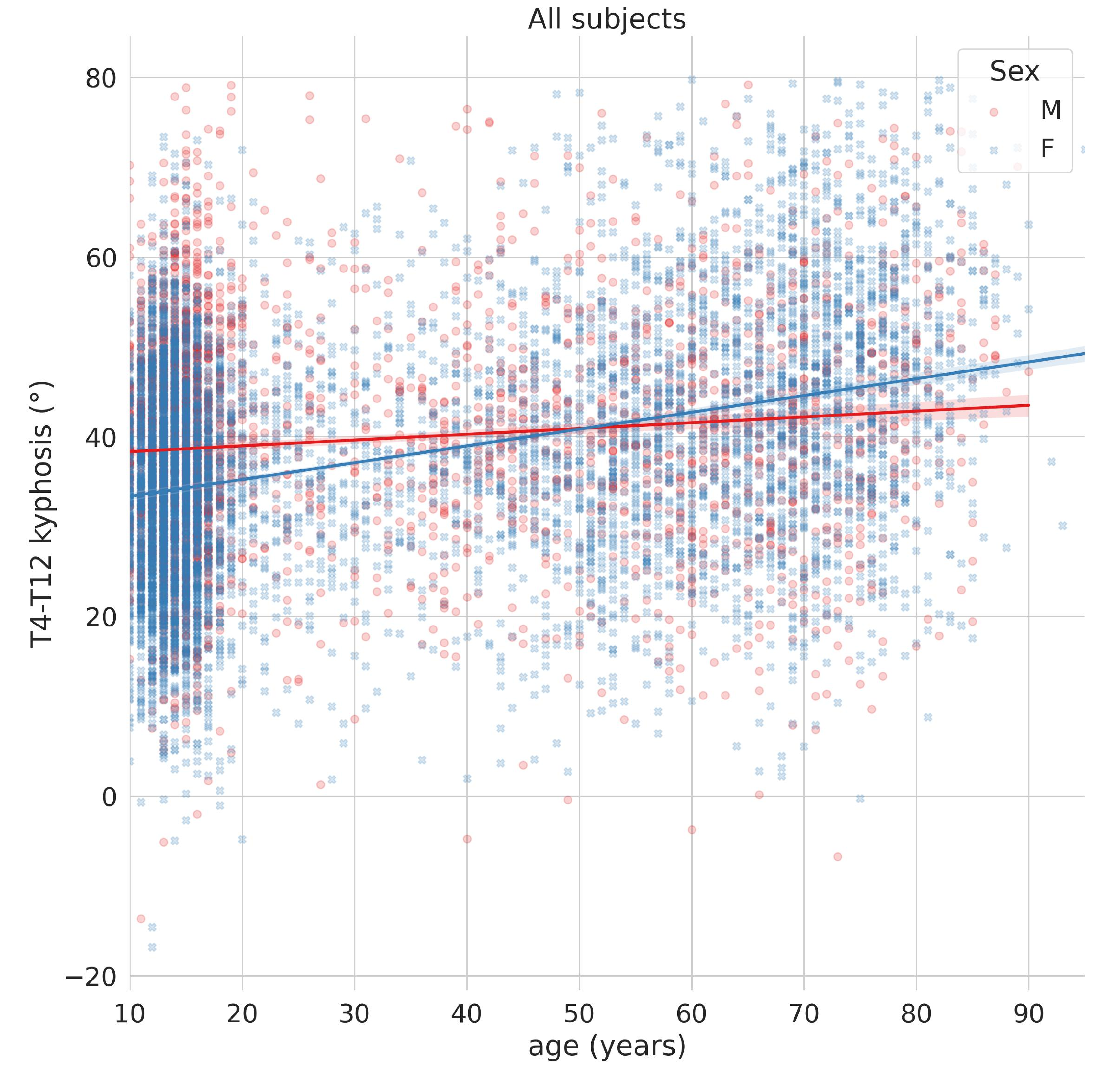
All subjects
Sex
M
F
T4-T12 kyphosis (°)
age (years)
80
60
40
20
0
−20
10
20
30
40
50
60
70
80
90

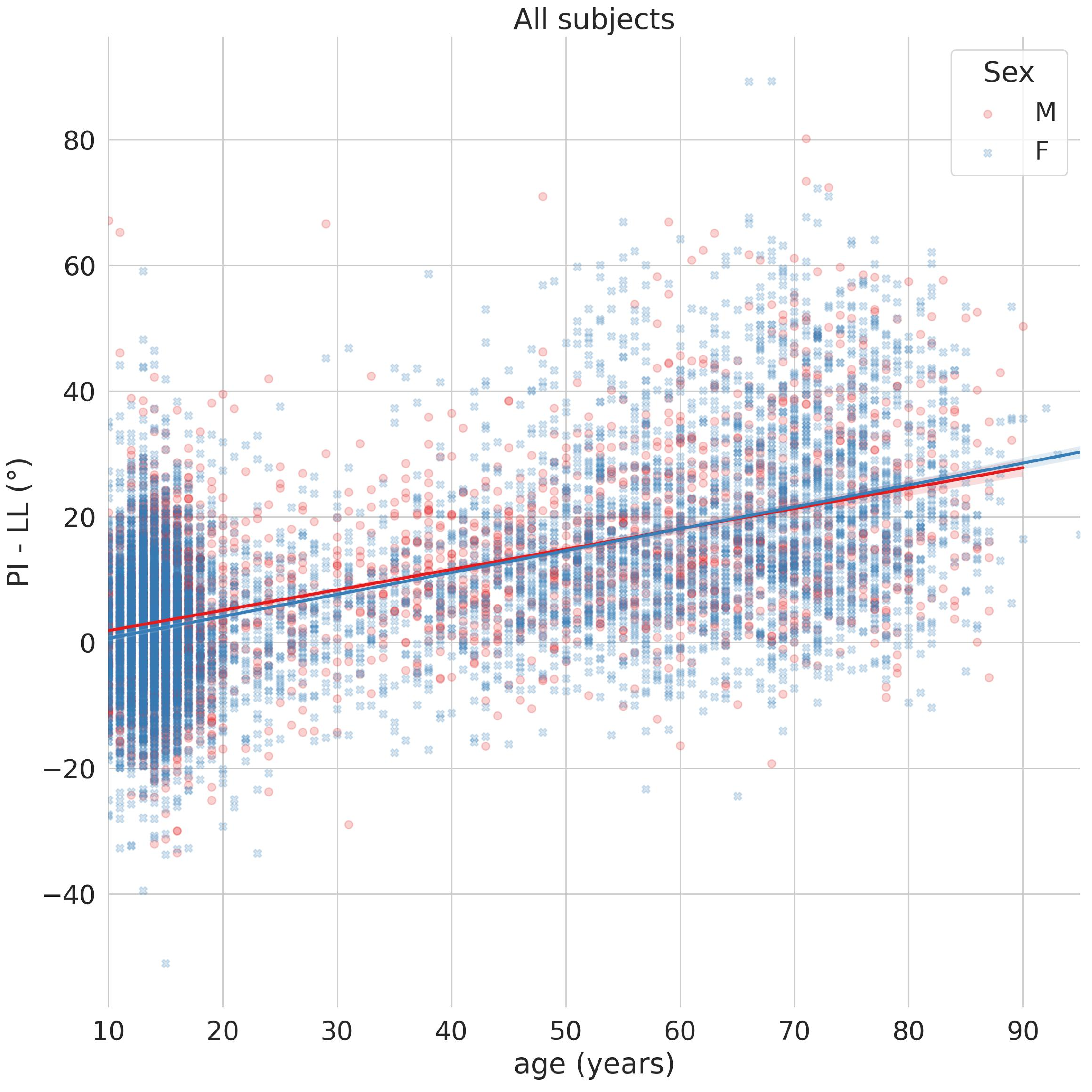

All subjects
Sex
M
F
PI - LL (°)
80
60
40
20
0
−20
−40
10
20
30
40
50
60
70
80
90
age (years)

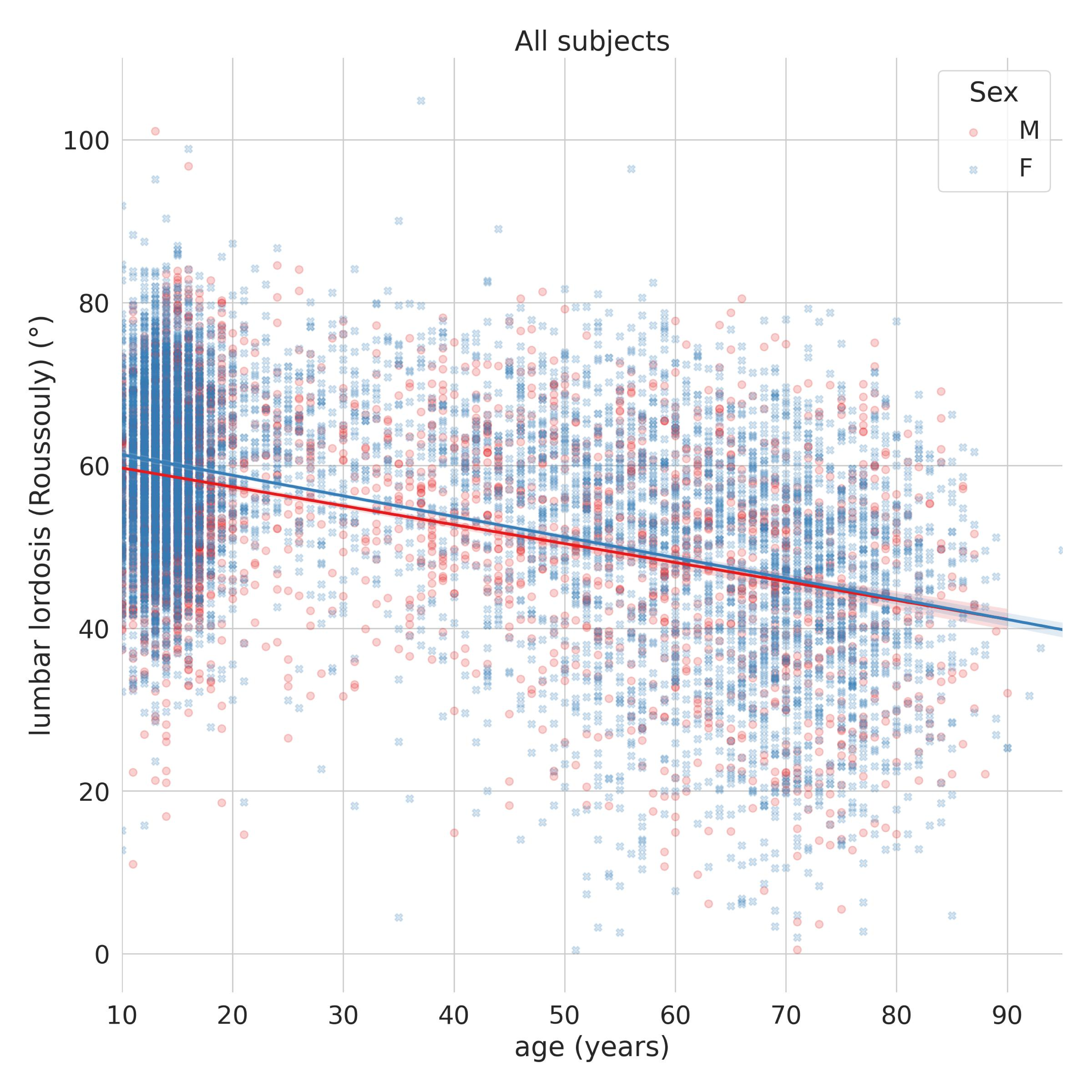

All subjects
Sex
M
F
lumbar lordosis (Roussouly) (°)
age (years)
0
20
40
60
80
100
10
20
30
40
50
60
70
80
90

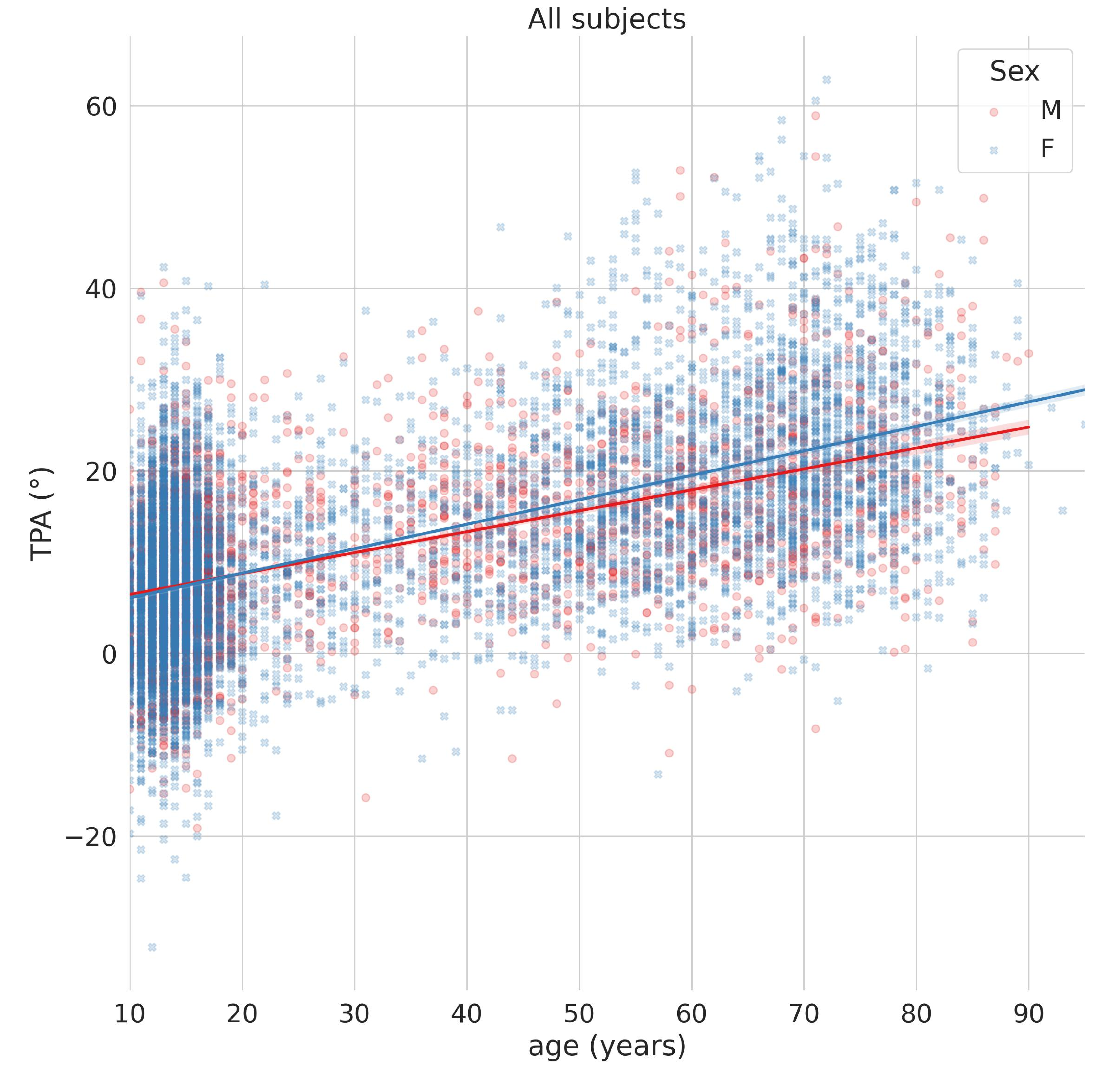
All subjects
Sex
M
F
60
40
20
0
−20
TPA (°)
10
20
30
40
50
60
70
80
90
age (years)

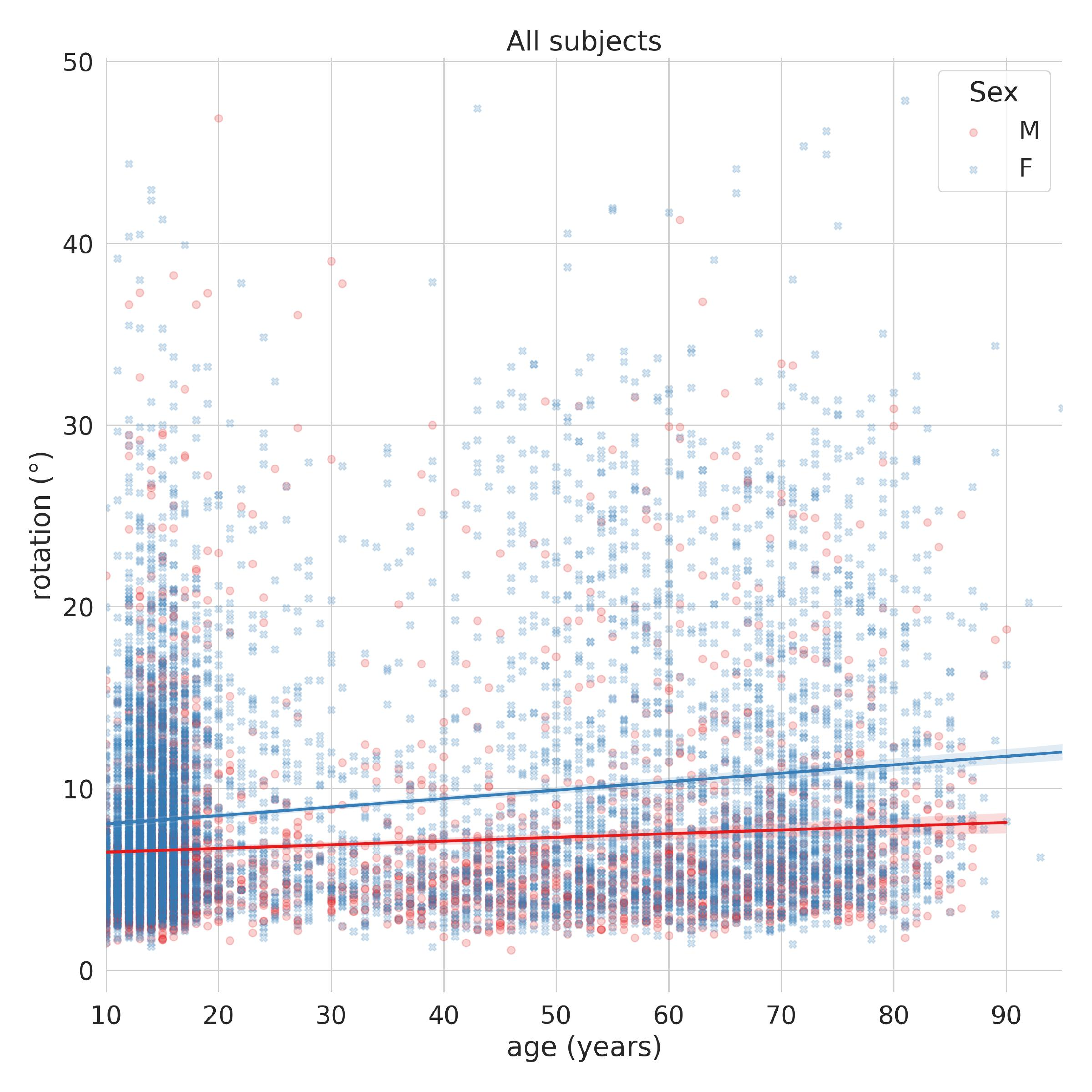
All subjects
Sex
M
F
rotation (°)
age (years)
0
10
20
30
40
50
10
20
30
40
50
60
70
80
90

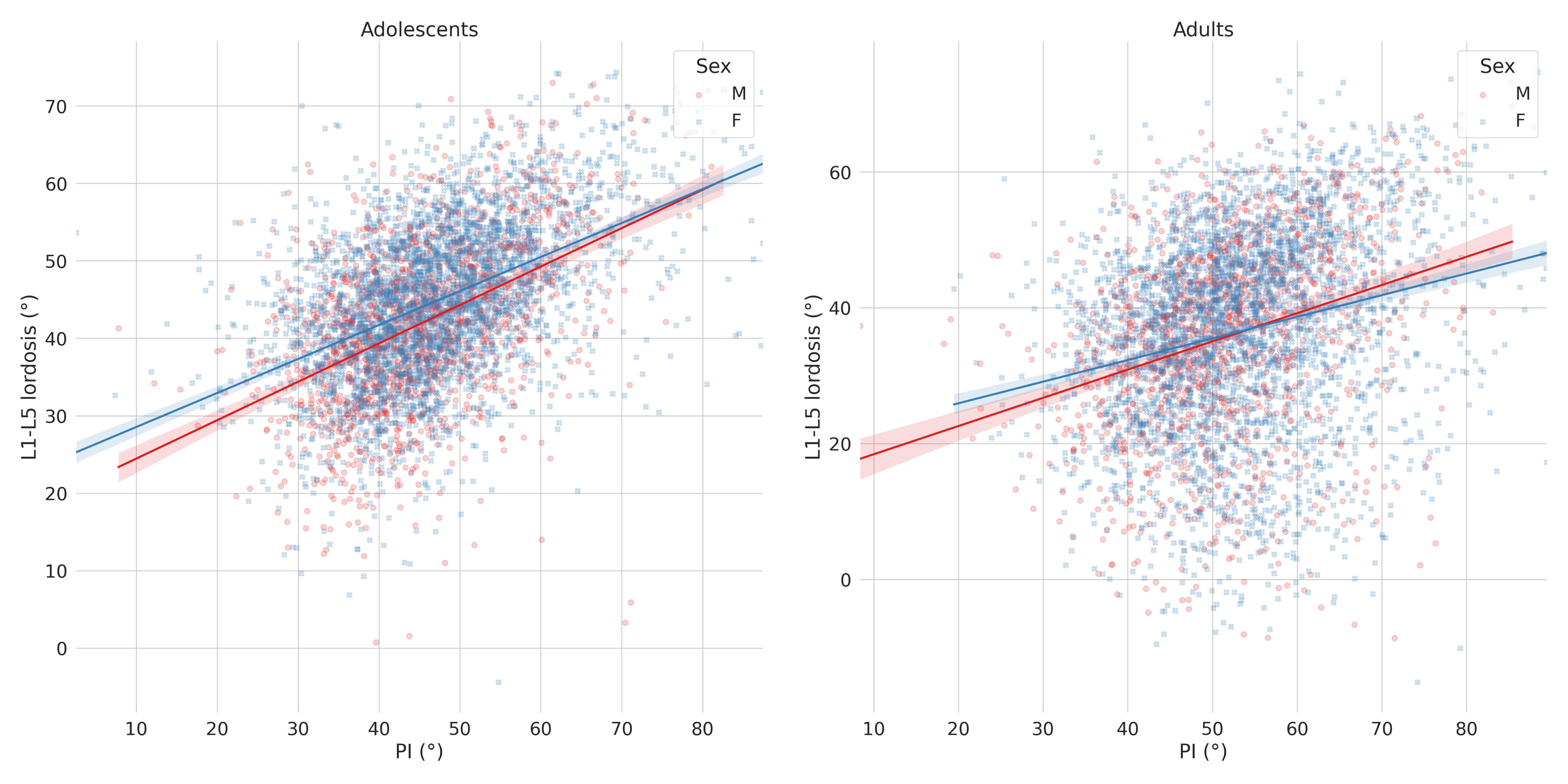
Adolescents
Adults
Sex
M
F
L1-L5 lordosis (°)
PI (°)

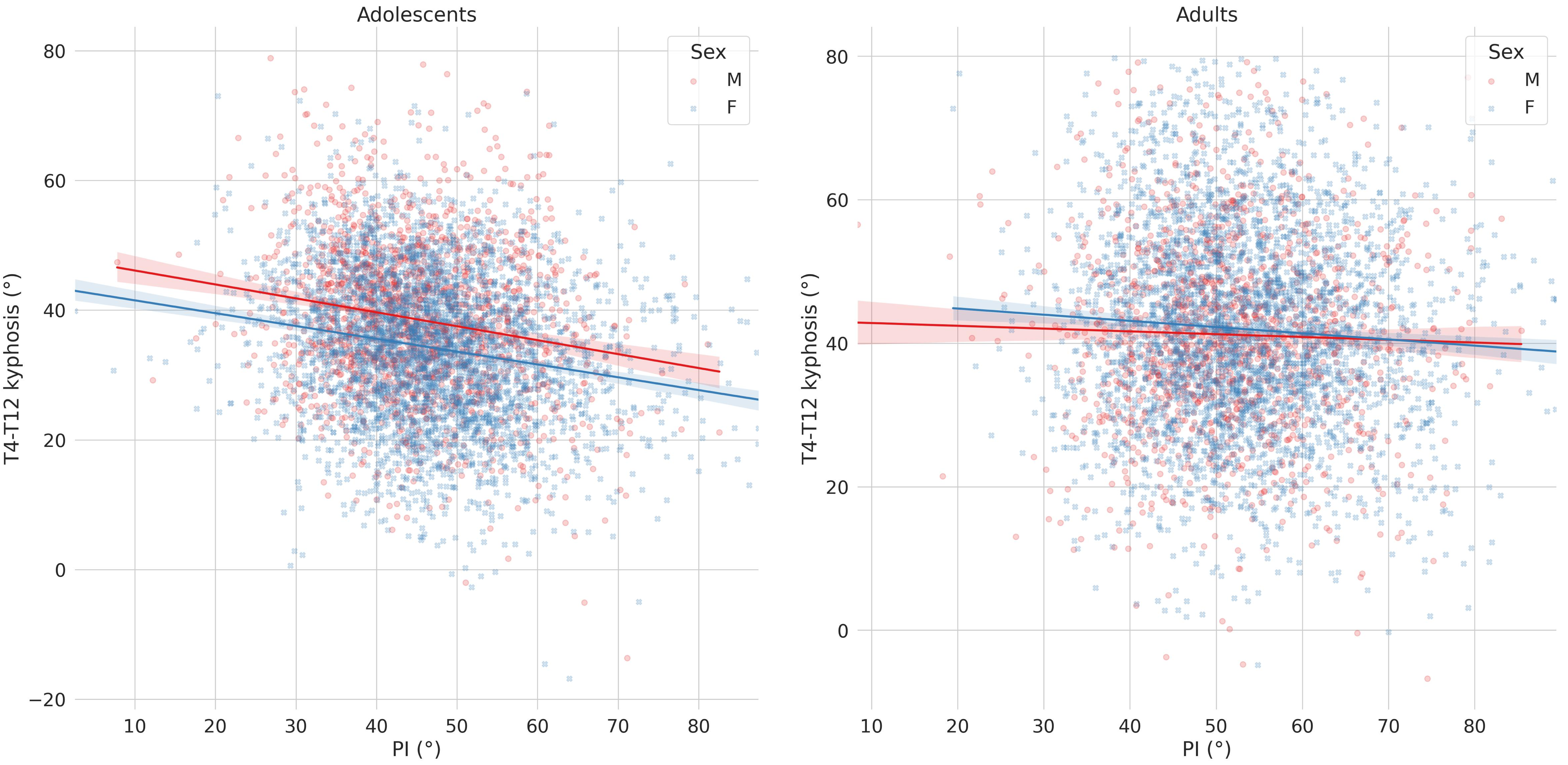

Adolescents
Adults
Sex
M
F
T4-T12 kyphosis (°)
PI (°)
80
60
40
20
0
−20
10
20
30
40
50
60
70
80

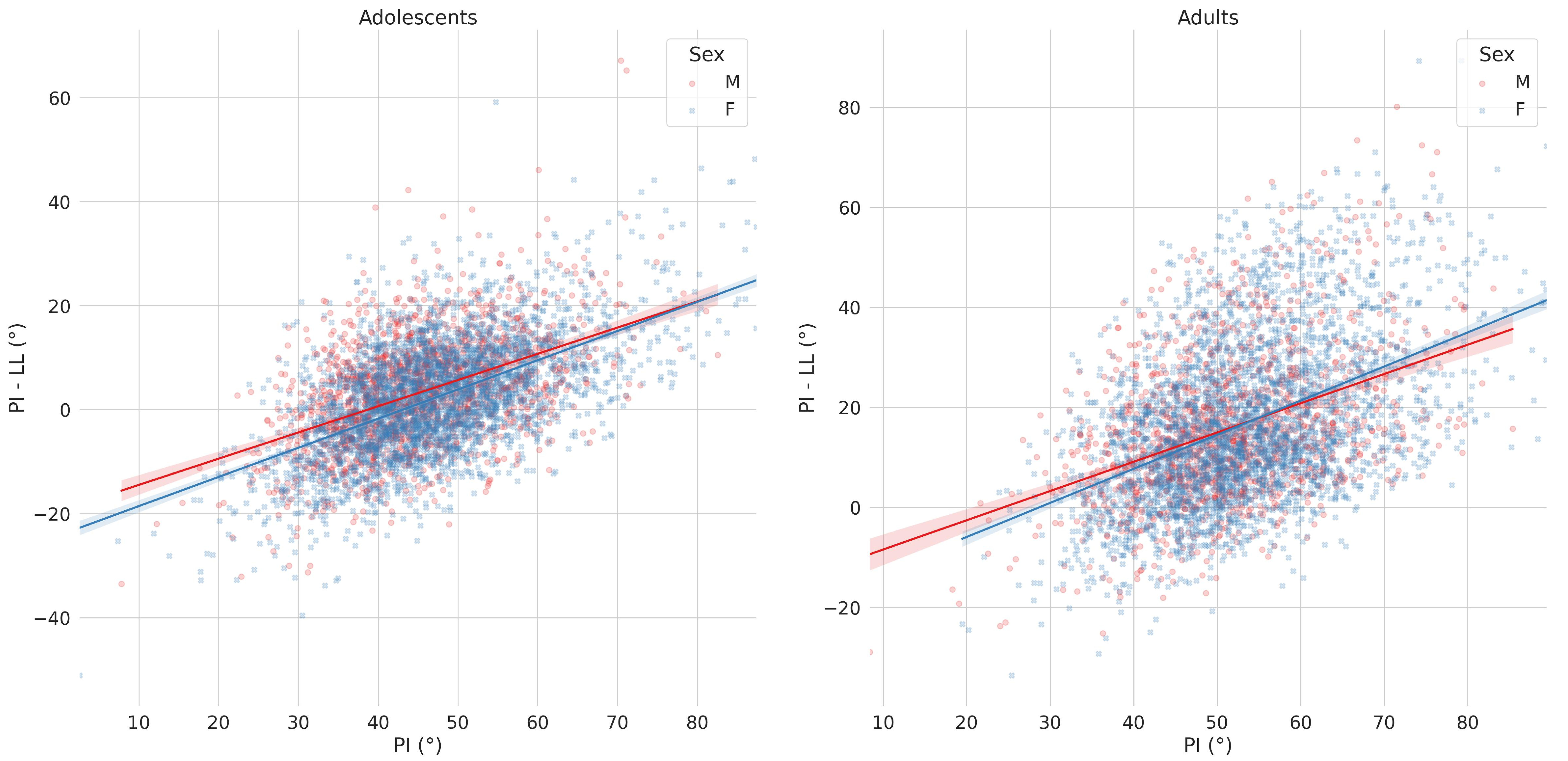

Adolescents
Adults
Sex
M
F
PI - LL (°)
PI (°)

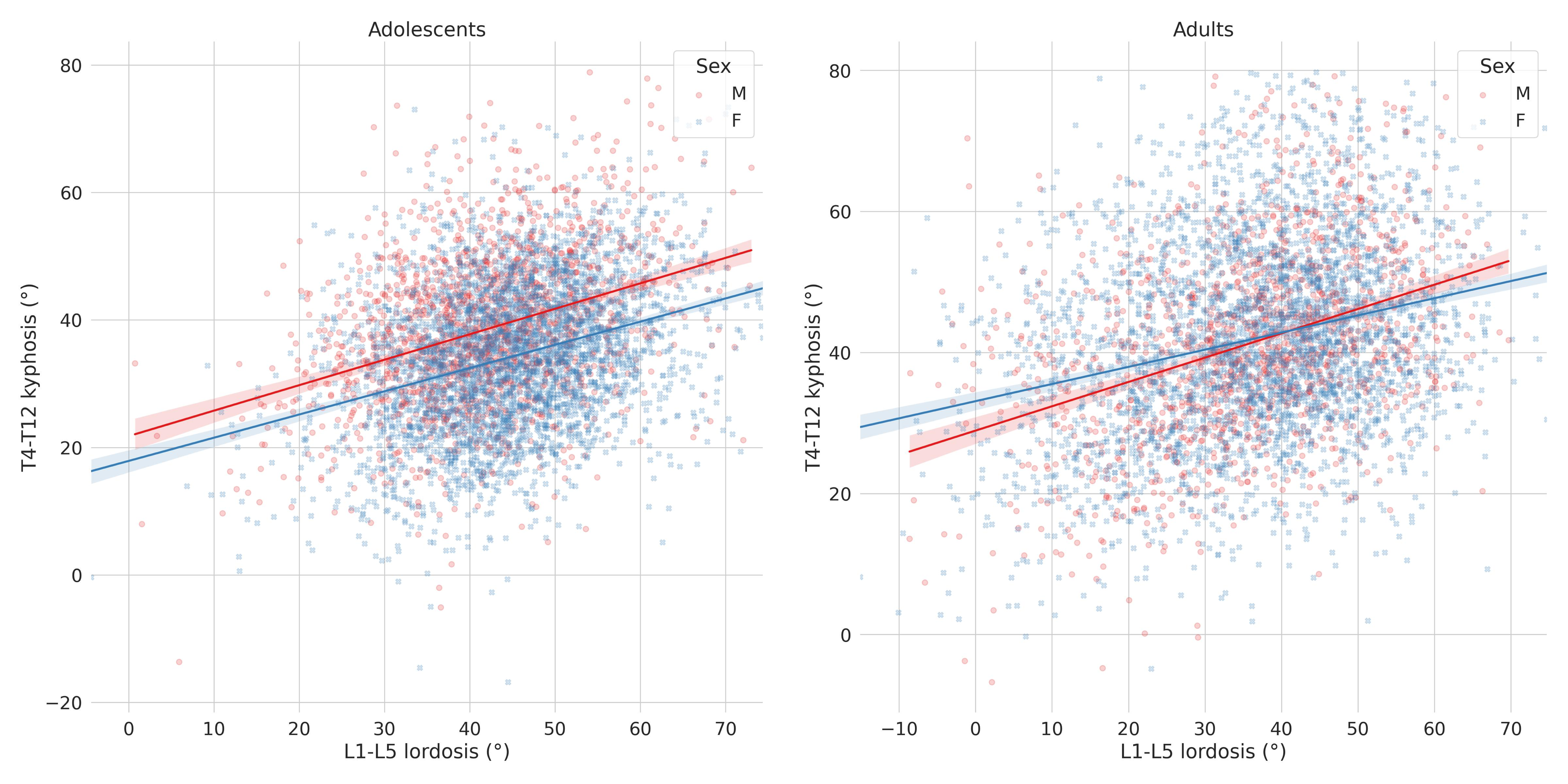
Adolescents
Adults
Sex
M
F
T4-T12 kyphosis (°)
L1-L5 lordosis (°)
80
60
40
20
0
−20
−10
10
30
50
70

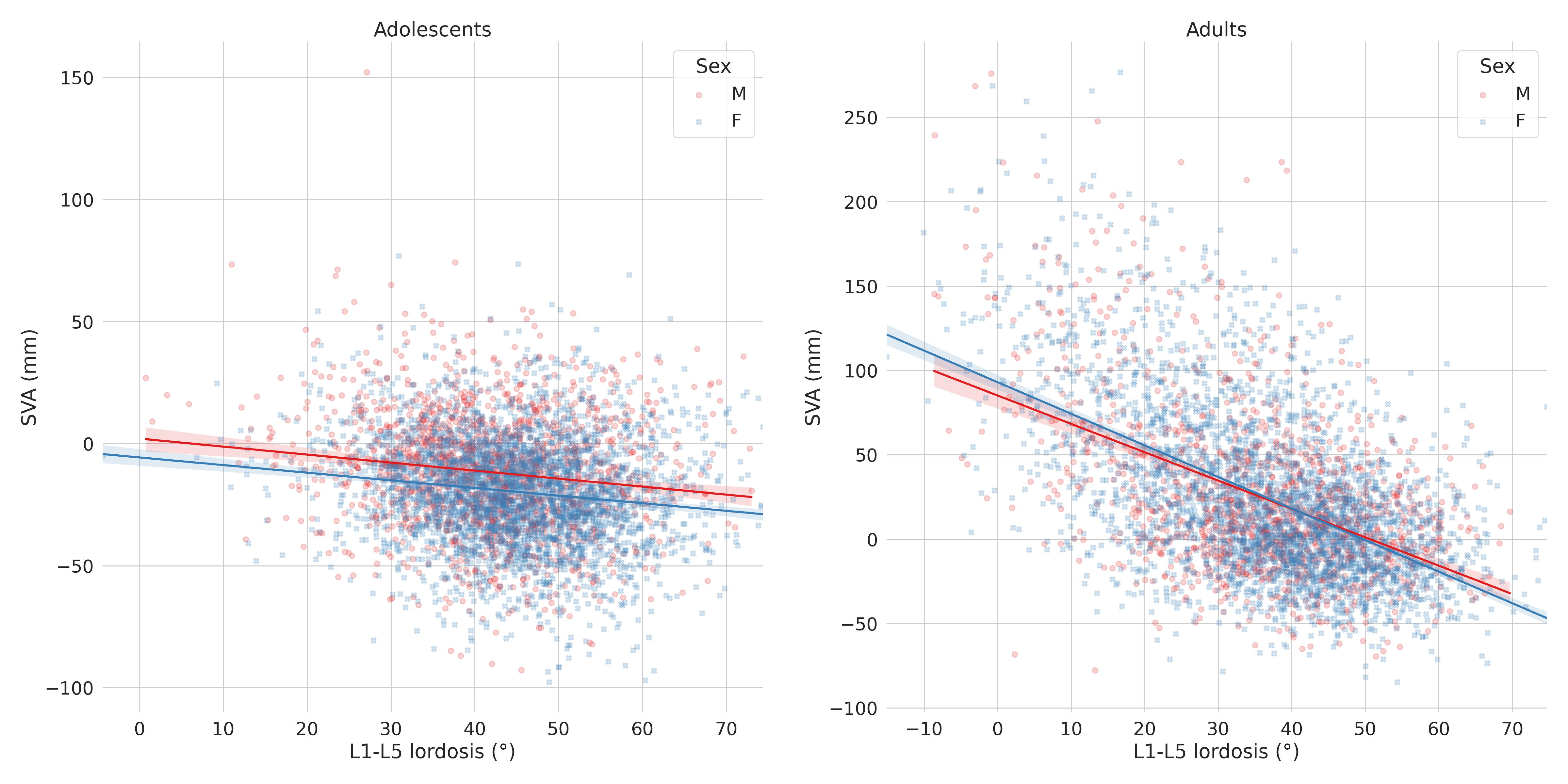

Adolescents
Adults
Sex
M
F
SVA (mm)
L1-L5 lordosis (°)
150
100
50
0
−50
−100
0
10
20
30
40
50
60
70
250
200
−10

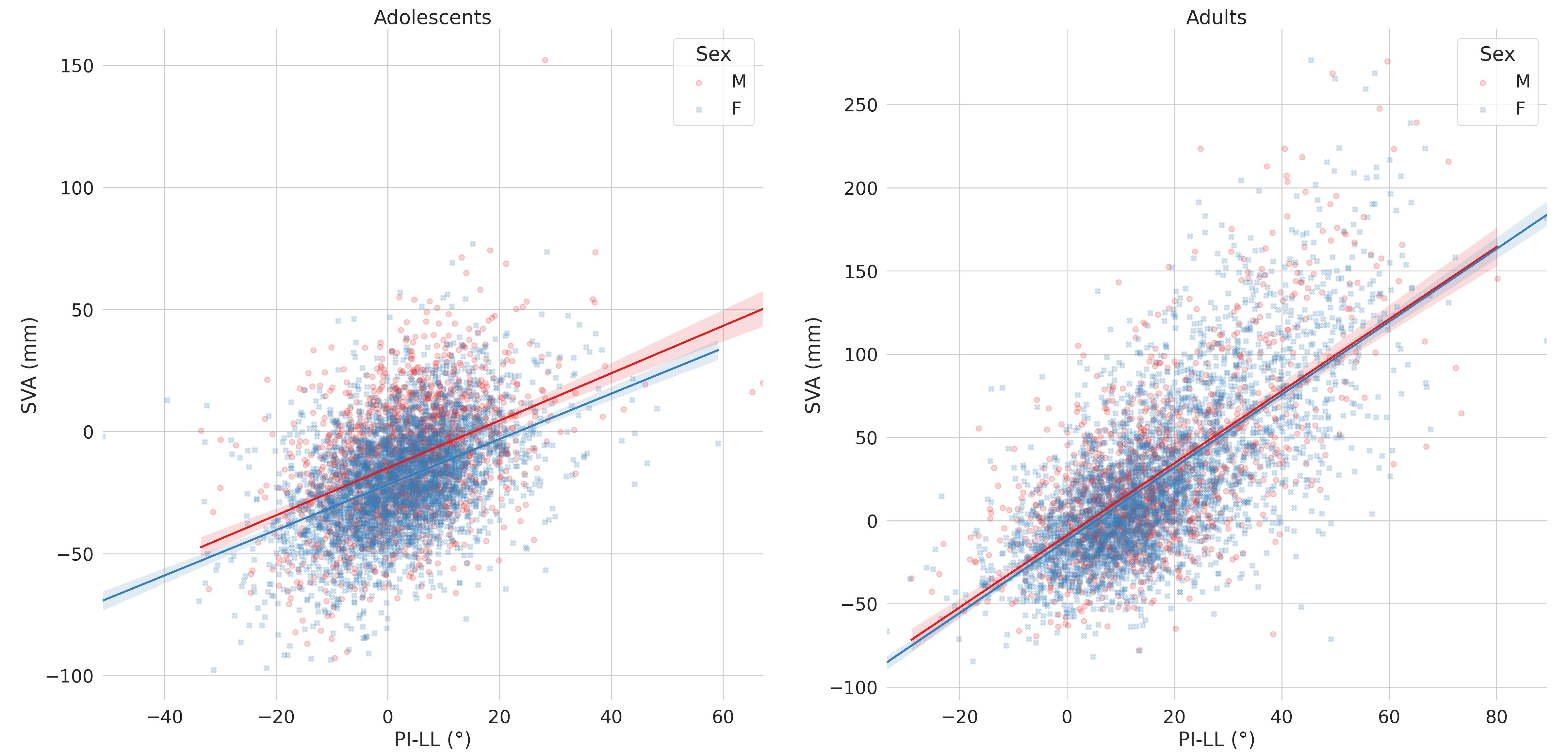

Adolescents
Adults
Sex
M
F
SVA (mm)
PI-LL (°)
150
100
50
0
−50
−100
−40
−20
0
20
40
60
250
200
80

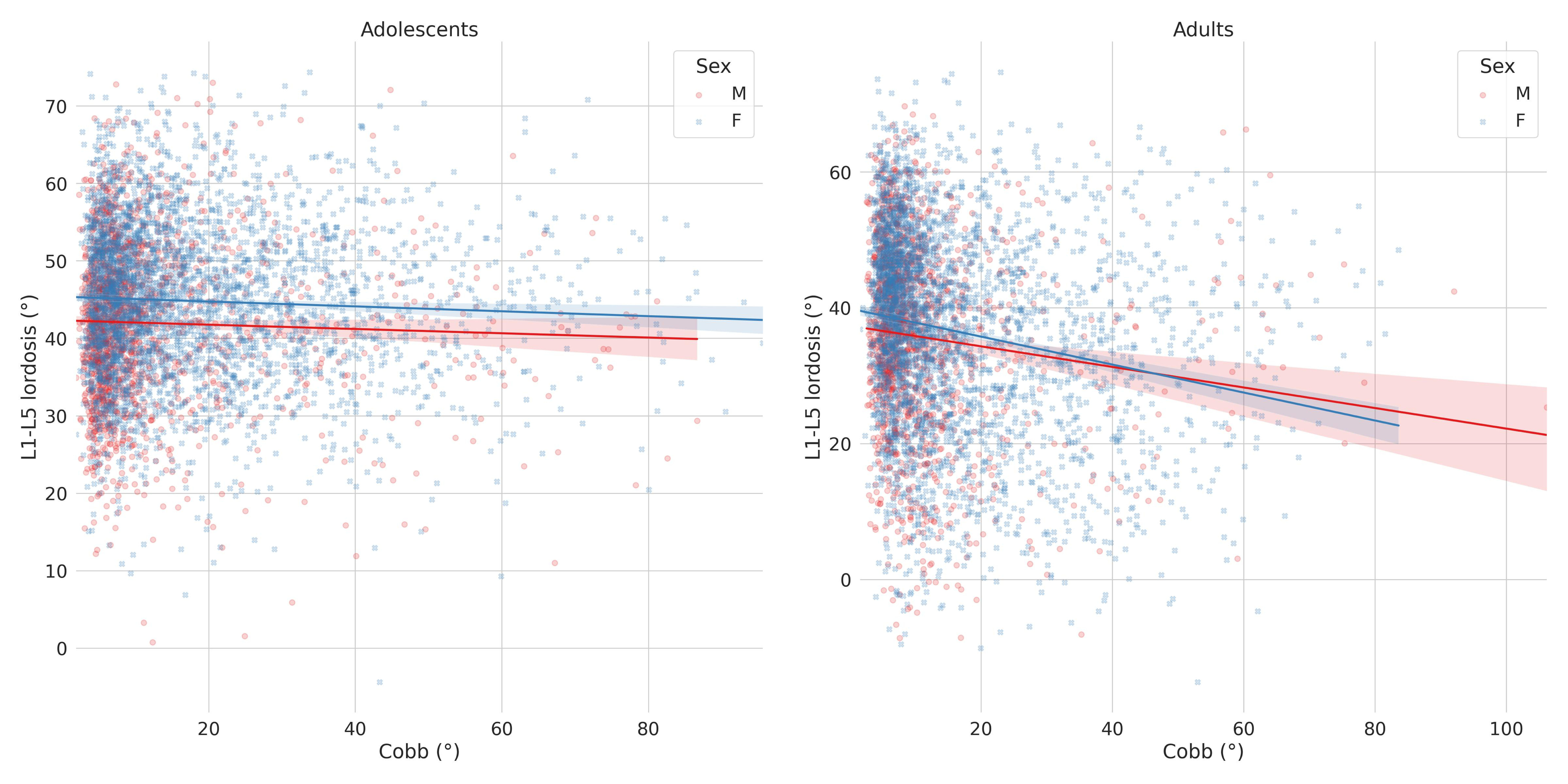

Adolescents
Adults
Sex
M
F
L1-L5 lordosis (°)
Cobb (°)
70
60
50
40
30
20
10
0
20
40
60
80
100

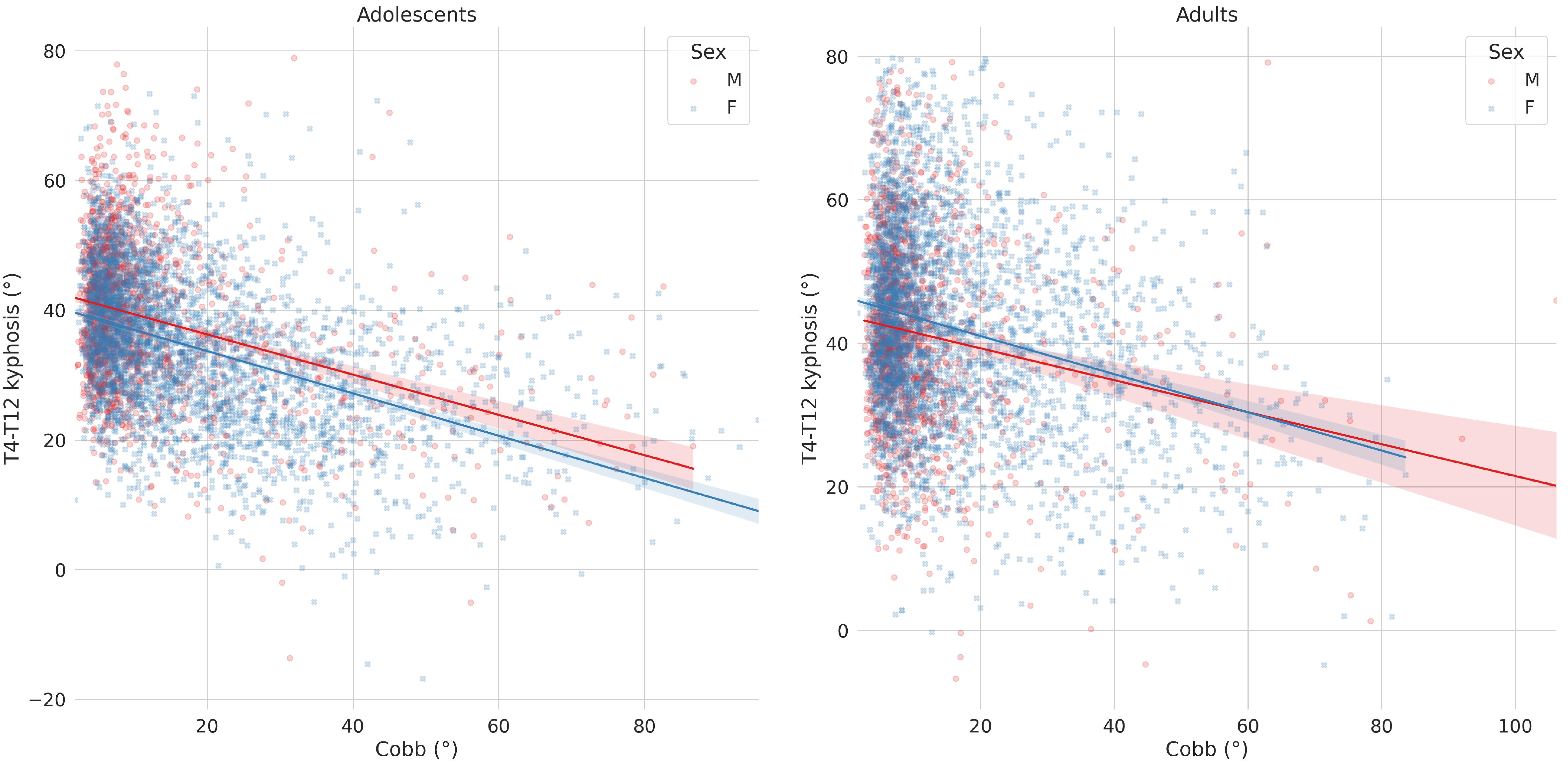
Adolescents
Adults
Sex
M
F
T4-T12 kyphosis (°)
Cobb (°)
80
60
40
20
0
−20
100

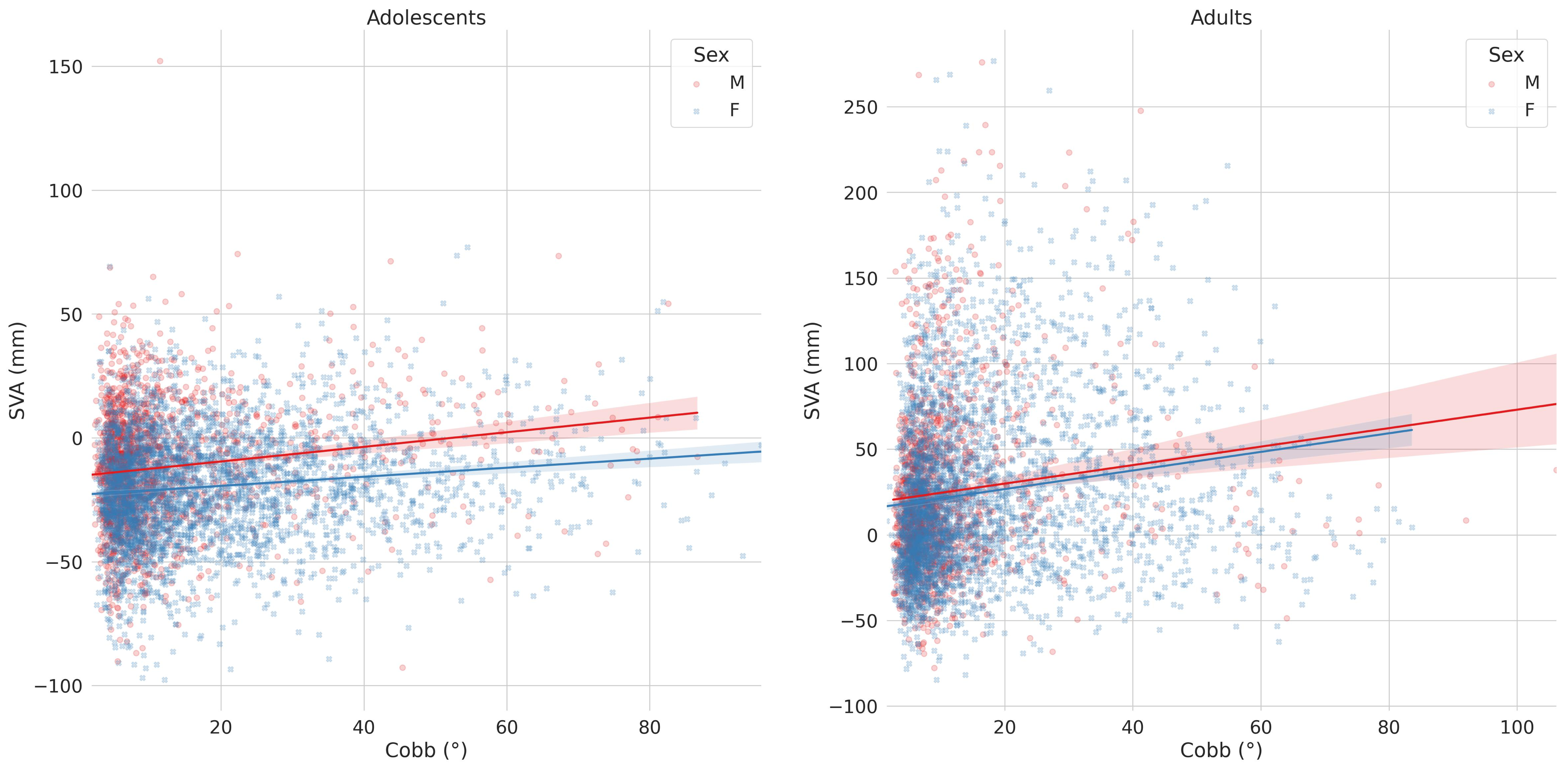

Adolescents
Adults
Sex
M
F
SVA (mm)
Cobb (°)
150
100
50
0
−50
−100
20
40
60
80
250
200
100
